# Supplementary material for: Impact of inconsistent ethnicity recordings on estimates of inequality in child health and education data: a data linkage study of Child and Adolescent Mental Health Services in South London
Source: BMJ Open. 2024 Mar 5;14(3):e078788. doi: 10.1136/bmjopen-2023-078788 (PMC10916132; doi:10.1136/bmjopen-2023-078788)
Supplement: Supplementary data [file bmjopen-2023-078788supp001.pdf]

Table S1: The RECORD statement – checklist of items, extended from the STROBE statement, that should be reported in observational studies using routinely collected health data. Items specific to cross-sectional/case-control studies have been removed.

	Item No.	STROBE items	Page	RECORD items	Page
**Title and abstract**					
	1	(a) Indicate the study's design with a commonly used term in the title or the abstract (b) Provide in the abstract an informative and balanced summary of what was done and what was found	2	RECORD 1.1: The type of data used should be specified in the title or abstract. When possible, the name of the databases used should be included.<br><br>RECORD 1.2: If applicable, the geographic region and timeframe within which the study took place should be reported in the title or abstract.<br><br>RECORD 1.3: If linkage between databases was conducted for the study, this should be clearly stated in the title or abstract.	2
**Introduction**					
Background rationale	2	Explain the scientific background and rationale for the investigation being reported	3-5	N/A	
Objectives	3	State specific objectives, including any prespecified hypotheses	3-5	N/A	
**Methods**					
Study Design	4	Present key elements of study design early in the paper	5	N/A	
Setting	5	Describe the setting, locations, and relevant dates, including periods of recruitment, exposure, follow-up, and data collection	5	N/A	
Participants	6	(a) Cohort study - Give the eligibility criteria, and the sources and methods of selection of participants. Describe methods of follow-up<br><br>(b) Cohort study - For matched studies, give matching criteria and number of exposed and unexposed	5	RECORD 6.1: The methods of study population selection (such as codes or algorithms used to identify subjects) should be listed in detail. If this is not possible, an explanation should be provided.<br><br>RECORD 6.2: Any validation studies of the codes or algorithms used to select the population should be referenced. If validation	5

	Item No.	STROBE items	Page	RECORD items	Page
				was conducted for this study and not published elsewhere, detailed methods and results should be provided.<br><br>RECORD 6.3: If the study involved linkage of databases, consider use of a flow diagram or other graphical display to demonstrate the data linkage process, including the number of individuals with linked data at each stage.	
Variables	7	Clearly define all outcomes, exposures, predictors, potential confounders, and effect modifiers. Give diagnostic criteria, if applicable.	6-8	RECORD 7.1: A complete list of codes and algorithms used to classify exposures, outcomes, confounders, and effect modifiers should be provided. If these cannot be reported, an explanation should be provided.	6-8
Data sources/ measurement	8	For each variable of interest, give sources of data and details of methods of assessment (measurement).<br>Describe comparability of assessment methods if there is more than one group	6-8	N/A	
Bias	9	Describe any efforts to address potential sources of bias	6-8	N/A	
Study size	10	Explain how the study size was arrived at	5	N/A	
Quantitative variables	11	Explain how quantitative variables were handled in the analyses. If applicable, describe which groupings were chosen, and why	N/A	N/A	
Statistical methods	12	(a) Describe all statistical methods, including those used to control for confounding<br>(b) Describe any methods used to examine subgroups and interactions<br>(c) Explain how missing data were addressed<br>(d) Cohort study - If applicable, explain how loss to follow-up was addressed<br>(e) Describe any sensitivity analyses	7-8	N/A	
Data access and cleaning methods		N/A		RECORD 12.1: Authors should describe the extent to which the investigators had access to the database population used to create the study population.	5, 20

	Item No.	STROBE items	Page	RECORD items	Page
				RECORD 12.2: Authors should provide information on the data cleaning methods used in the study.	
Linkage		N/A		RECORD 12.3: State whether the study included person-level, institutional-level, or other data linkage across two or more databases. The methods of linkage and methods of linkage quality evaluation should be provided.	5
**Results**					
Participants	13	(a) Report the numbers of individuals at each stage of the study (e.g., numbers potentially eligible, examined for eligibility, confirmed eligible, included in the study, completing follow-up, and analysed)<br>(b) Give reasons for non-participation at each stage.<br>(c) Consider use of a flow diagram	Supplement	RECORD 13.1: Describe in detail the selection of the persons included in the study (i.e., study population selection) including filtering based on data quality, data availability and linkage. The selection of included persons can be described in the text and/or by means of the study flow diagram.	5
Descriptive data	14	(a) Give characteristics of study participants (e.g., demographic, clinical, social) and information on exposures and potential confounders<br>(b) Indicate the number of participants with missing data for each variable of interest<br>(c) Cohort study - summarise follow-up time (e.g., average and total amount)	9	N/A	
Outcome data	15	Cohort study - Report numbers of outcome events or summary measures over time	9	N/A	
Main results	16	(a) Give unadjusted estimates and, if applicable, confounder-adjusted estimates and their precision (e.g., 95% confidence interval). Make clear which confounders were adjusted for and why they were included<br>(b) Report category boundaries when continuous variables were categorized<br>(c) If relevant, consider translating estimates of relative risk into absolute risk for a meaningful time period	11-14	N/A	

	Item No.	STROBE items	Page	RECORD items	Page
Other analyses	17	Report other analyses done—e.g., analyses of subgroups and interactions, and sensitivity analyses	12	N/A	
**Discussion**					
Key results	18	Summarise key results with reference to study objectives	15-17	N/A	
Limitations	19	Discuss limitations of the study, taking into account sources of potential bias or imprecision. Discuss both direction and magnitude of any potential bias	17-18	RECORD 19.1: Discuss the implications of using data that were not created or collected to answer the specific research question(s). Include discussion of misclassification bias, unmeasured confounding, missing data, and changing eligibility over time, as they pertain to the study being reported.	17-18
Interpretation	20	Give a cautious overall interpretation of results considering objectives, limitations, multiplicity of analyses, results from similar studies, and other relevant evidence	15-17	N/A	
Generalisability	21	Discuss the generalisability (external validity) of the study results	18	N/A	
**Other Information**					
Funding	22	Give the source of funding and the role of the funders for the present study and, if applicable, for the original study on which the present article is based	19	N/A	
Accessibility of protocol, raw data, and programming code		N/A		RECORD 22.1: Authors should provide information on how to access any supplemental information such as the study protocol, raw data, or programming code.	20

*Reference: Benchimol, E. I., Smeeth, L., Guttmann, A., Harron, K., Moher, D., Petersen, I., ... & RECORD Working Committee. (2015). The REporting of studies Conducted using Observational Routinely-collected health Data (RECORD) statement. PLoS Med, 12(10), e1001885.

*Checklist is protected under Creative Commons Attribution (CC BY) license.

Figure S1: Study flow diagram.

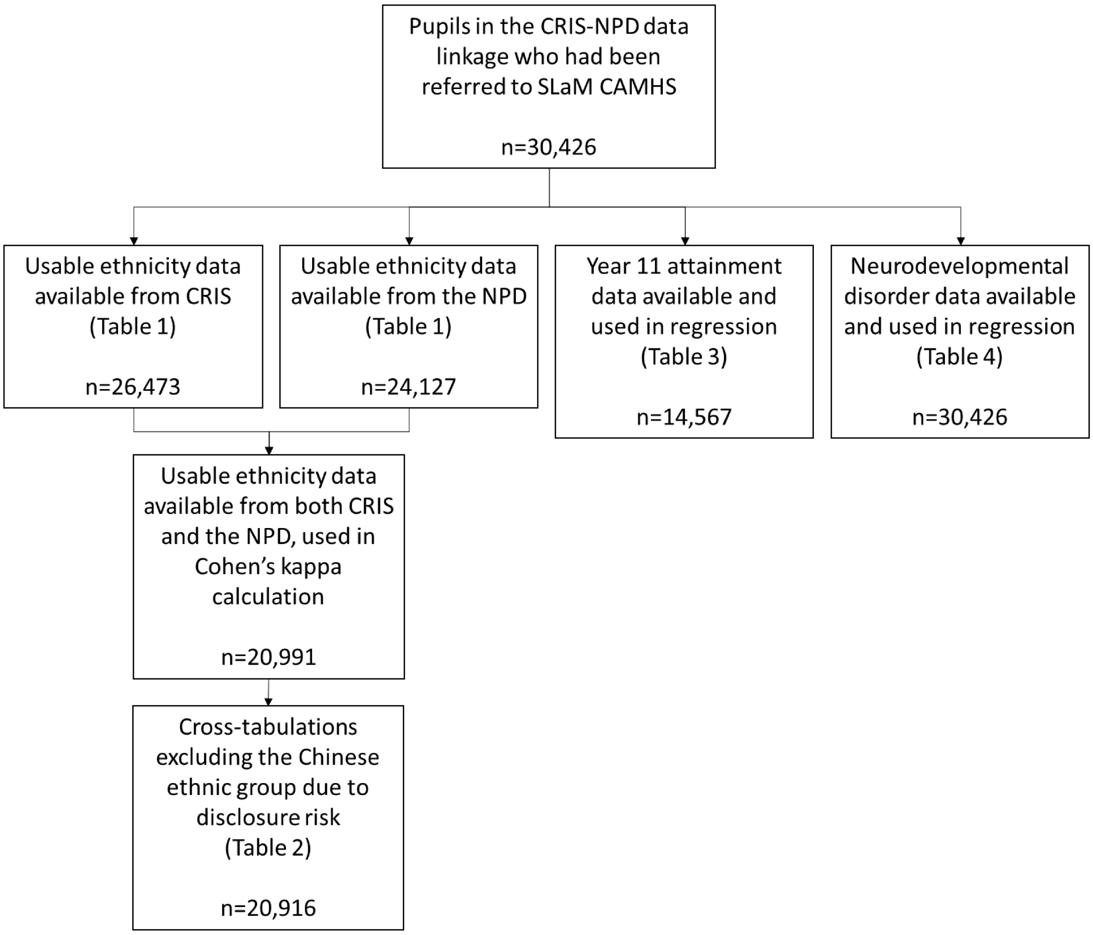

Table S2: Ethnic groups in the NPD.

Approved extended categories	Minor ethnic group	Major ethnic group
White – British	White - British	White
White – Cornish [a]		
White – English		
White – Scottish		
White – Welsh		
Other White British		
White – Irish	White	
Traveller of Irish heritage		
Any other White background	Any other White background	
Albanian		
Bosnian-Herzegovinian		
Croatian		
Greek/Greek Cypriot		
Greek		
Greek Cypriot		
Italian [a]		
Kosovan		
Portuguese		
Serbian		
Turkish/Turkish Cypriot		
Turkish		
Turkish Cypriot		
White European [a]		
White Eastern European [a]		
White Western European [a]		
White other		
Gypsy/Roma	Gypsy/Roma	
Gypsy		
Roma		
Other Gypsy/Roma		
White and Black Caribbean	White and Black Caribbean	Mixed/Dual background
White and Black African	White and Black African	
White and Asian	White and Asian	
White and Pakistani [a]		
White and Indian [a]		
White and any other Asian background		
Any other Mixed background	Any other Mixed background	
Asian and any other ethnic group [a]		
Asian and Black		
Asian and Chinese		
Black and any other ethnic group [a]		
Black and Chinese		

Approved extended categories	Minor ethnic group	Major ethnic group
Chinese and any other ethnic group [a]		
White and any other ethnic group [a]		
White and Chinese		
Other Mixed background		
Indian	Indian	Asian or Asian British
Pakistani	Pakistani	
Mirpuri Pakistani [a]		
Kashmiri Pakistani [a]		
Other Pakistani		
Bangladeshi	Bangladeshi	
Any other Asian background	Any other Asian background	
African Asian [a]		
Kashmiri other [a]		
Nepali [a]		
Sri Lankan Sinhalese		
Sri Lankan Tamil		
Sri Lankan other		
Other Asian		
Black Caribbean	Black Caribbean	Black or Black British
Black - African	Black - African	
Black - Angolan		
Black - Congolese [a]		
Black – Ghanaian		
Black – Nigerian		
Black - Sierra Leonean [a]		
Black – Somali		
Black – Sudanese		
Other Black African		
Any other Black background	Any other Black background	
Black European [a]		
Black North American [a]		
Other Black		
Chinese	Chinese	Chinese
Hong Kong Chinese [a]		
Malaysian Chinese		
Singaporean Chinese [a]		
Taiwanese [a]		
Other Chinese		
Any other ethnic group	Any other ethnic group	Any other ethnic group
Afghan [a]		
Arab other		
Egyptian [a]		
Filipino		
Iranian		

Approved extended categories	Minor ethnic group	Major ethnic group
Iraqi		
Japanese		
Korean [a]		
Kurdish		
Latin/South/Central American [a]		
Lebanese [a]		
Libyan [a]		
Malay		
Moroccan [a]		
Polynesian [a]		
Thai [a]		
Vietnamese		
Yemeni		
Other ethnic group		
Refused	Refused	Refused
Information not yet obtained	Information not yet obtained	Information not yet obtained

a. Specific ethnicities not included in the SLaM electronic health record ethnic categories list (CRIS, Supplement 4).

Note: Contains public sector information licensed under the Open Government Licence v3.0.

Table S3: Ethnic groups in CRIS.

SLaM electronic health record ethnic categories	16+1 ethnic categories supplied from CRIS
British (A)	British (A)
English (CA)	
Scottish (CB)	
Welsh (CC)	
Irish (B)	Irish (B)
Irish Traveller (CL)	
Albanian (CS)	Any other White background €
All former USSR Republics (CQ) [a]	
Bosnian (CT)	
Croatian (CU)	
Cypriot (part not stated) (CE) [a]	
Greek (CF)	
Greek Cypriot (CG)	
Gypsy/Romany (CN)	
Kosovan (CR)	
Kurdish (C5)	
Other former Yugoslavia (CW) [a]	
Other White Unspecified (C3)	
Other White/Mixed European (CY) [a]	
Portuguese (C4)	
Serbian (CV)	
Traveller (CM) [a]	
Turkish (CH)	
Turkish Cypriot (CJ)	
White and Black Caribbean (D)	White and Black Caribbean (D)
White and Black African €	White and Black African €
White and Asian (F)	White and Asian (F)
Asian and Chinese (GE)	Any other Mixed background (G)
Black and Asian (GA)	
Black and Chinese (GB)	
Black and White (GC) [a]	
Chinese and White (GD)	
Indian/British Indian (H)	Indian (H)
Pakistani/British Pakistani (J)	Pakistani (J)
Bangladeshi/British Bangladeshi (K)	Bangladeshi (K)
British Asian (LH) [a]	Any other Asian background (L)
Caribbean Asian (LJ) [a]	
East African Asian (LD) [a]	
Mixed Asian (LA) [a]	
Other Asian Unspecified (LK)	
Sinhalese (LG)	
Sri Lankan (LE)	
Tamil (LF)	

SLaM electronic health record ethnic categories	16+1 ethnic categories supplied from CRIS
Caribbean (M)	Caribbean (M)
Algerian (PP) [a]<br>Angolan (PJ)<br>Eritrean (PK) [a]<br>Ethiopian (PL) [a]<br>Ghanaian (PM)<br>Nigerian (PC)<br>Somali (PA)<br>Sudanese (PH)<br>Ugandan (PQ) [a]<br>Other African (N)	African (N)
Mixed Black (PB) [a]<br>Black British (PD) [a]<br>Other Black Unspecified (PE)	Any other Black background (P)
Chinese (R)	Chinese (R)
Any Other Group (SE)<br>Arab (SG)<br>Columbian (SK) [a]<br>Ecuadorian (SL) [a]<br>Filipino (SC)<br>Iranian (SH)<br>Iraqi (SJ)<br>Japanese (SB)<br>Malaysian (SD)<br>Middle Eastern (SF) [a]<br>Other Latin American (SM)<br>Vietnamese (SA)	Any other ethnic group (S)
Not Stated (Z)	Not Stated (Z)

a. Specific ethnicities not included in the 'approved extended ethnic categories' list (NPD, Supplement 3).

Table S4: Aggregating CRIS ethnicity to assist comparison with the NPD.

16+1 ethnic categories supplied from CRIS	Aggregate category
British (A)<br>Irish (B)<br>Any other White background (C)	White
White and Black Caribbean (D)<br>White and Black African (E)<br>White and Asian (F)<br>Any other Mixed background (G)	Mixed
Indian (H)<br>Pakistani (J)<br>Bangladeshi (K)<br>Any other Asian background (L)	Asian
Caribbean (M)<br>African (N)<br>Any other Black background (P)	Black
Chinese (R)	Chinese
Any other ethnic group (S)	Other
Not Stated (Z)	Unknown

Table S5: Proportion of disaggregated CRIS ethnicities categorised as consistent major ethnic groups in the NPD, among individuals where ethnicity was available from both sources (n=20,916).

		NPD				
		**White**	**Black**	**Asian**	**Mixed**	**Other**
**CRIS**	**British**	94.6%				
	**Irish**	93.8%				
	**Any other White background**	80.5%				
	**African**		91.6%			
	**Caribbean**		90.2%			
	**Any other Black background**		84.3%			
	**Indian**			83.5%		
	**Pakistani**			89.9%		
	**Bangladeshi**			94.6%		
	**Any other Asian background**			55.5%		
	**White and Asian**				77.6%	
	**White and Black African**				75.2%	
	**White and Black Caribbean**				79.2%	
	**Any other Mixed background**				74.3%	
	**Any other ethnic group**					26.7%

Note: Row percentages provided. Off-diagonal proportions have been suppressed to avoid disclosive cell counts. For the purposes of this table, we have excluded pupils who were categorised as Chinese in either data source, in order to avoid disclosive cell counts.

Table S6: Unadjusted risk ratios between ethnicity derived from either NPD or CRIS (exposure) and whether the Year 11 expected attainment threshold was achieved (outcome; 'no' is the reference group)

	NPD-derived ethnicity as exposure, supplemented from CRIS if missing				CRIS-derived ethnicity as exposure, supplemented from NPD if missing			
	**No (%)**	**Yes (%)**	**RR**	**95% CI**	**No (%)**	**Yes (%)**	**RR**	**95% CI**
**White**	5,641 (67.8%)	2,682 (32.2%)	Reference	-	5,725 (67.4%)	2,765 (32.6%)	Reference	-
**Black**	2,420 (73.2%)	885 (26.8%)	0.83	0.78 to 0.89	2,473 (73.5%)	891 (26.5%)	0.81	0.76 to 0.87
**Asian**	311 (61.1%)	198 (38.9%)	1.21	1.07 to 1.34	317 (62.4%)	191 (37.6%)	1.15	1.02 to 1.29
**Chinese**	25 (53.2%)	22 (46.8%)	1.45	1.03 to 1.88	18 (40.9%)	26 (59.1%)	1.81	1.35 to 2.22
**Mixed**	1,002 (67.7%)	478 (32.3%)	1.00	0.92 to 1.08	758 (69.1%)	339 (30.9%)	0.95	0.86 to 1.04
**Other**	280 (63.9%)	158 (36.1%)	1.12	0.98 to 1.26	388 (64.8%)	211 (35.2%)	1.08	0.96 to 1.21
**Missing**	330 (71.0%)	135 (29.0%)	0.90	0.78 to 1.04	330 (71.0%)	135 (29.0%)	0.89	0.77 to 1.03

Note: n=14,567. The n=15,859 not included in these analyses were missing data on Year 11 attainment. The n=817 in the missing ethnicity group are those with ethnicity data available neither in CRIS, nor the NPD.

Abbreviations: CI=Confidence Interval, CRIS=Clinical Record Interactive Search, NPD=National Pupil Database, RR=Risk Ratio.

Table S7: Unadjusted risk ratios between ethnicity derived from either NPD or CRIS (exposure) and neurodevelopmental disorder diagnosis (outcome; 'no' is the reference group)

	NPD-derived ethnicity as exposure, supplemented from CRIS if missing				CRIS-derived ethnicity as exposure, supplemented from NPD if missing			
	**No (%)**	**Yes (%)**	**RR**	**95% CI**	**No (%)**	**Yes (%)**	**RR**	**95% CI**
**White**	11,098 (69.5%)	4,880 (30.5%)	Reference	-	11,377 (69.7%)	4,947 (30.3%)	Reference	-
**Black**	5,896 (74.3%)	2,039 (25.7%)	0.84	0.80 to 0.88	6,017 (73.9%)	2,129 (26.1%)	0.86	0.82 to 0.90
**Asian**	782 (75.5%)	254 (24.5%)	0.80	0.71 to 0.89	816 (75.4%)	266 (24.6%)	0.81	0.72 to 0.90
**Chinese**	74 (76.3%)	23 (23.7%)	0.77	0.53 to 1.09	69 (79.3%)	18 (20.7%)	0.68	0.44 to 1.01
**Mixed**	2,635 (71.3%)	1,060 (28.7%)	0.94	0.89 to 0.99	1,941 (71.1%)	790 (28.9%)	0.95	0.89 to 1.02
**Other**	711 (81.9%)	157 (18.1%)	0.59	0.51 to 0.68	976 (78.8%)	263 (21.2%)	0.70	0.62 to 0.78
**Missing**	660 (80.8%)	157 (19.2%)	0.62	0.54 to 0.72	660 (80.8%)	157 (19.2%)	0.63	0.54 to 0.73

Note: n=30,426. The n=817 in the missing ethnicity group are those with ethnicity data available neither in CRIS, nor the NPD.
Abbreviations: CI=Confidence Interval, CRIS=Clinical Record Interactive Search, NPD=National Pupil Database, RR=Risk Ratio.

Table S8: Ethnicity overview after supplementing missing ethnicity data from one source with ethnicity data from the other source.

		n (%)
**Usable NPD-derived ethnicity, supplemented from CRIS if missing, n=29,609**		
	White	15,978 (54.0%)
	Black	7,935 (26.8%)
	Asian	1,036 (3.5%)
	Chinese	97 (0.3%)
	Mixed	3,695 (12.5%)
	Other	868 (2.9%)
**Usable CRIS-derived ethnicity, supplemented from the NPD if missing, n=29,609**		
	White	16,324 (55.1%)
	Black	8,146 (27.5%)
	Asian	1,082 (3.7%)
	Chinese	87 (0.3%)
	Mixed	2,731 (9.2%)
	Other	1,239 (4.2%)
