# Supplementary material for: Impact of inconsistent ethnicity recordings on estimates of inequality in child health and education data: a data linkage study of Child and Adolescent Mental Health Services in South London
Source: BMJ Open. 2024 Mar 5;14(3):e078788. doi: 10.1136/bmjopen-2023-078788 (PMC10916132; doi:10.1136/bmjopen-2023-078788)
Supplement: Author's manuscript [file bmjopen-2023-078788.draft_revisions.pdf]

BMJ Open

BMJ Open is committed to open peer review. As part of this commitment we make the peer review history of every article we publish publicly available.

When an article is published we post the peer reviewers' comments and the authors' responses online. We also post the versions of the paper that were used during peer review. These are the versions that the peer review comments apply to.

The versions of the paper that follow are the versions that were submitted during the peer review process. They are not the versions of record or the final published versions. They should not be cited or distributed as the published version of this manuscript.

BMJ Open is an open access journal and the full, final, typeset and author-corrected version of record of the manuscript is available on our site with no access controls, subscription charges or pay-per-view fees (http://bmjopen.bmj.com).

If you have any questions on BMJ Open's open peer review process please email info.bmjopen@bmj.com

BMJ Open

The impact of inconsistent ethnicity recordings on estimates of inequality in child health and education data: A data linkage study

Journal:	*BMJ Open*
Manuscript ID	bmjopen-2023-078788
Article Type:	Original research
Date Submitted by the Author:	11-Aug-2023

Complete List of Authors:	Wickersham, Alice; King's College London, CAMHS Digital Lab, Department of Child and Adolescent Psychiatry<br>Das-Munshi, Jayati; King's College London, Psychological Medicine<br>Ford, Tamsin; University of Cambridge, Psychiatry<br>Jewell, Amelia; South London and Maudsley NHS Foundation Trust, Maudsley Biomedical Research Centre<br>Stewart, Robert; King's College London, Department of Psychological Medicine<br>Downs, Johnny; King's College London, CAMHS Digital Lab, Department of Child and Adolescent Psychiatry

Keywords:	MENTAL HEALTH, Health Equity, EDUCATION & TRAINING (see Medical Education & Training), EPIDEMIOLOGIC STUDIES, Child & adolescent psychiatry < PSYCHIATRY

SCHOLARONE™
Manuscripts

I, the Submitting Author has the right to grant and does grant on behalf of all authors of the Work (as defined in the below author licence), an exclusive licence and/or a non-exclusive licence for contributions from authors who are: i) UK Crown employees; ii) where BMJ has agreed a CC-BY licence shall apply, and/or iii) in accordance with the terms applicable for US Federal Government officers or employees acting as part of their official duties; on a worldwide, perpetual, irrevocable, royalty-free basis to BMJ Publishing Group Ltd ("BMJ") its licensees and where the relevant Journal is co-owned by BMJ to the co-owners of the Journal, to publish the Work in this journal and any other BMJ products and to exploit all rights, as set out in our licence.

The Submitting Author accepts and understands that any supply made under these terms is made by BMJ to the Submitting Author unless you are acting as an employee on behalf of your employer or a postgraduate student of an affiliated institution which is paying any applicable article publishing charge ("APC") for Open Access articles. Where the Submitting Author wishes to make the Work available on an Open Access basis (and intends to pay the relevant APC), the terms of reuse of such Open Access shall be governed by a Creative Commons licence – details of these licences and which Creative Commons licence will apply to this Work are set out in our licence referred to above.

Other than as permitted in any relevant BMJ Author's Self Archiving Policies, I confirm this Work has not been accepted for publication elsewhere, is not being considered for publication elsewhere and does not duplicate material already published. I confirm all authors consent to publication of this Work and authorise the granting of this licence.

For peer review only

The impact of inconsistent ethnicity recordings on estimates of inequality in child health and education data: A data linkage study

Alice Wickersham [a*], Jayati Das-Munshi [b], Tamsin Ford [c], Amelia Jewell [d], Robert Stewart [b] and Johnny Downs [a]

[a] CAMHS Digital Lab, Department of Child and Adolescent Psychiatry, King's College London, London, United Kingdom; [b] Department of Psychological Medicine, King's College London, London, United Kingdom; [c] Department of Psychiatry, University of Cambridge, Cambridge, United Kingdom; [d] South London and Maudsley NHS Foundation Trust, London, United Kingdom.

* Corresponding author. CAMHS Digital Lab, Department of Child and Adolescent Psychiatry, Institute of Psychiatry, Psychology and Neuroscience, King's College London, 16 De Crespigny Park, London, SE5 8AF

alice.wickersham@kcl.ac.uk

ORCID: 0000-0002-7402-7690

Word count: 3090

For peer review only

Abstract

Objectives: Ethnicity data are critical for identifying inequalities, but previous studies suggest ethnicity is not consistently recorded between different administrative datasets. With researchers increasingly leveraging cross-domain data linkages, we investigated the completeness and consistency of ethnicity data in two linked health and education datasets.

Design: Cohort study.

Setting: South London and Maudsley NHS Foundation Trust de-identified electronic health records, accessed via Clinical Record Interactive Search (CRIS), and the National Pupil Database (NPD) (2007-2013).

Participants: n=30,426 children and adolescents with linked CRIS and NPD records.

Primary and secondary outcome measures: Ethnicity data were compared between CRIS and the NPD. Associations between ethnicity as recorded from each source and key educational and clinical outcomes were explored with logistic regression.

Results: Ethnicity data were available for 79.3% from the NPD, 87.0% from CRIS, 97.3% from either source and 69.0% from both sources. Among those who had ethnicity data from both, the two data sources agreed on 87.0% of aggregate ethnicity categorisations overall, but with high levels of disagreement in Mixed and Other ethnic groups. Strengths of associations between ethnicity, educational attainment and neurodevelopmental disorder varied according to which data source was used to code ethnicity. For example, as compared to White pupils, a significantly higher proportion of Asian pupils achieved expected educational attainment thresholds only if ethnicity was coded from the NPD (OR=1.85, 95% CI=1.48 to 2.31, p<0.001), not if ethnicity was coded from CRIS (OR=1.18, 95% CI=0.97 to 1.44, p=0.099).

Conclusions: Data linkage has the potential to minimise missing ethnicity data, and overlap in ethnicity categorisations between CRIS and the NPD was generally high. However, choosing which data source to primarily code ethnicity from can have implications for analyses of ethnicity, mental health, and educational outcomes. Users of linked data should exercise caution in combining and comparing ethnicity between different data sources.

Key words: Ethnicity, Mental Health, Education, Data Linkage, Administrative Data

For peer review only - http://bmjopen.bmj.com/site/about/guidelines.xhtml

Article summary: Strengths and limitations of this study

- We leveraged a data linkage between routinely-collected health and education datasets for a large cohort of South London children and adolescents.
- We conducted a comprehensive analysis of the availability and consistency of ethnicity data between these health and education datasets.
- Only one assessment of ethnicity was available from each data source and for each individual, precluding analysis of within-person changes in identity over time.
- Aggregate ethnicity data were primarily used for analysis.
- Nonetheless, this study highlights important discrepancies in ethnicity data with implications for inequalities research.

Introduction

Ethnicity data are critical for highlighting inequalities in the population and informing public health and educational policies. For example, some minority ethnic groups are thought to perform less well in school assessments,[1-3] and to be underdiagnosed with neurodevelopmental disorders.[4] A recent review reported significant ethnic inequalities in access, experiences and outcomes of healthcare in the United Kingdom, but also highlighted that very few national datasets have sufficiently high quality ethnicity data to robustly investigate these inequalities.[5] Incomplete and inconsistent ethnicity data in administrative datasets could result in variable estimates for important clinical and educational outcomes, compromising our ability to identify and address these inequalities.

Ethnicity is a fluid construct which should be self-ascribed and is socially constructed, capturing shared characteristics such as geography, ancestry, culture and language.[6] Epidemiological studies using routinely collected data often rely on ethnicity variables which have been aggregated into several top-level categories. This is due to small sample sizes in more granular ethnic groups, associated concerns with statistical power, and the risk of

For peer review only

For peer review only - http://bmjopen.bmj.com/site/about/guidelines.xhtml

inadvertent de-anonymisation which arises when sharing detailed ethnicity data. However, aggregating ethnicity data results in problematic within-group heterogeneity, and risks specific ethnic groups being overlooked in research.[6-8] Not only do these aggregate classifications fail to meaningfully capture homogenous groups, but they also do not necessarily align across different data sources. Previous research from the United States of America and United Kingdom has repeatedly raised concerns over the completeness and consistency of ethnicity variables in routinely collected health data, particularly in comparison to self-reported survey data.[9-12]

A recent United Kingdom report on Hospital Episode Statistics, the Emergency Care Data Set, and the Community Services Dataset found that ethnicity recordings in these datasets were often incomplete, and that for minority ethnic groups in particular, ethnicity codes were not used consistently for the same individuals over time.[13] Another United Kingdom study compared two linked administrative health datasets from primary care and hospital settings, and found that combining the data sources increased the completeness of ethnicity data.[14] While agreement between these sources was generally high, with 85% of patients having the same ethnicity recorded in both primary care and hospital data, agreement was much weaker for South Asian and Other ethnic groups.

We sought to explore this issue in linked administrative data from health and education domains. With cross-disciplinary data linkages increasingly being adopted for epidemiological research, an understanding of the completeness and consistency of ethnicity from two such data sources would be beneficial. The aims of this exploratory analysis were therefore to (1) summarise the completeness of ethnicity data in the linked health and education records; (2) summarise the alignment of aggregate ethnicity between the two data sources; and (3) investigate whether key educational and clinical outcomes vary according to which data source is used to code ethnicity.

For peer review only - http://bmjopen.bmj.com/site/about/guidelines.xhtml

Materials and methods

Data

We used an existing data linkage between de-identified electronic health records from South London and Maudsley NHS Foundation Trust (SLaM), and educational records from the Department for Education. SLaM serves the boroughs of Croydon, Lambeth, Lewisham and Southwark, and provides some national services. The SLaM catchment area is ethnically diverse, and has a lower proportion of individuals from White ethnic backgrounds compared to London and England as a whole.[15] SLaM makes de-identified electronic health records available for research via the Clinical Record Interactive Search (CRIS) system. For children and adolescents (aged 4 to 18 years) referred to SLaM Child and Adolescent Mental Health Services (CAMHS) between 2007 and 2013, individual-level data linkage was undertaken between CRIS and the National Pupil Database (NPD).[16,17] This analysis focused on pupils in the CRIS-NPD data linkage who had been referred to SLaM CAMHS, either from within the SLaM catchment area or from out-of-area (n=30,426). Reporting follows RECORD guidelines (Table S1 and Figure S1).

The CRIS data resource, including the linked data used in this manuscript, has received research ethics approval for secondary analyses (Oxford REC C, reference 18/SC/0372). The CRIS Oversight Committee ensures that research conducted using the health records are ethical and legal, and service users can opt-out of their CRIS data being used for research.

Patient and public involvement statement

The data linkage used in this study was carried out in consultation with several patient and caregiver groups at the National Institute for Health Research Biomedical Research Centre, South London and Maudsley National Health Service Foundation Trust and King's College London. This has been described elsewhere.[16,17]

For peer review only - http://bmjopen.bmj.com/site/about/guidelines.xhtml

Ethnicity – NPD

In the NPD, pupil ethnicity is collected as part of the school census undertaken by England's state schools. It is either self-ascribed or parent-ascribed, although historically this information could also be ascribed by the school.[18] Due to concerns over sensitivity and potential disclosure, ethnicity extracts from the NPD are sometimes supplied to researchers as what is termed 'major ethnic groups' only (Table S2). Major ethnic groups are similar to the aggregated groups used by the Office for National Statistics; however, Chinese is provided as a separate group rather than aggregated under Asian/Asian British (White, Black, Asian, Chinese, Mixed, Other, and Unknown).[19]

Ethnicity – CRIS

In SLaM source data, accessed via CRIS, patient ethnicity is recorded in structured fields and intended to be self-ascribed, although in practice, ethnicity may sometimes be assumed and ascribed by the clinician or other staff member completing the record. CRIS aggregates ethnicity recorded in structured fields into 16+1 categories (Table S3), and this is the format that the ethnicity variable is made available to researchers. For the pupils in this study, ethnicity was extracted from electronic health records via CRIS in March 2020.

To assist comparison with the NPD, we further aggregated CRIS ethnicity into White, Black, Asian, Chinese, Mixed, Other and Unknown (Table S4), although it should be noted that some inconsistencies between the resulting aggregate ethnic groups remain. Most notably, the NPD places Kurdish groups into the Other major ethnic group, while CRIS places them in the 16+1 category "any other White background", such that they were subsequently aggregated as White in CRIS. Also, the NPD places Malaysian groups into the Chinese major ethnic group, while CRIS places them in the 16+1 category "any other ethnic group", such that they were subsequently aggregated as Other in CRIS. Due to the aforementioned concerns around data security and confidentiality, we were unable to access

For peer review only - http://bmjopen.bmj.com/site/about/guidelines.xhtml

more granular ethnicity variables to make these categorisations consistent between the data sources.

Statistical analysis

First, we summarised the completeness of ethnicity variables in the NPD and CRIS. We initially did this including the Unknown categories, then excluding these categories to determine the availability of what a previous study termed 'usable ethnicity'.[14] Second, we used descriptive statistics and Cohen's kappa to explore how usable ethnicity compared between the NPD and CRIS for those who had ethnicity variables available from both data sources.[20,21]

Finally, we conducted bivariate logistic regression analyses to estimate how ethnicity from each data source was associated with the outcome variables of educational attainment in School Year 11 (achieving expected attainment threshold of five A* to C General Certificates of Secondary Education [GCSEs] or equivalent grades including English and maths, derived from the NPD) and neurodevelopmental disorder (any record of an intellectual disability, pervasive developmental disorder, or hyperkinetic disorder, derived from CRIS structured primary and secondary diagnosis fields). We focused on neurodevelopmental disorders because they are often pervasive and lifelong,[22] therefore overcoming biases which might arise in differential timing of diagnoses. In order to understand how missing ethnicity data in either source might introduce bias into analyses of these variables, we combined all Unknown labels and empty cells into a single 'Missing' category. Analyses were conducted in Stata 15.1.[23]

For peer review only - http://bmjopen.bmj.com/site/about/guidelines.xhtml

Results

Completeness of ethnicity variables

Including the Unknown categories, NPD ethnicity was available for n=25,951 (85.3%), and CRIS ethnicity was available for n=28,363 (93.2%). Excluding the Unknown categories, usable NPD ethnicity was available for n=24,127 (79.3%), and usable CRIS ethnicity was available for n=26,473 (87.0%) (Table 1). In total, n=29,609 (97.3%) had usable ethnicity data from either the NPD or from CRIS, and n=20,991 (69.0%) had usable ethnicity data from both sources.

Table 1: Sample overview.

		n (%)
**Usable NPD-derived ethnicity, n=24,127**		
	White	13,259 (55.0%)
	Black	6,278 (26.0%)
	Asian	755 (3.1%)
	Chinese	73 (0.3%)
	Mixed	3,247 (13.5%)
	Other	515 (2.1%)
**Usable CRIS-derived ethnicity, n=26,473**		
	White	14,572 (55.0%)
	Black	7,400 (28.0%)
	Asian	959 (3.6%)
	Chinese	82 (0.3%)
	Mixed	2,294 (8.7%)
	Other	1,166 (4.4%)
**Year 11 expected attainment threshold achieved, n=14,567**		
	No	10,009 (68.7%)
	Yes	4,558 (31.3%)
**Neurodevelopmental disorder diagnosis, n=30,426**		
	No	21,856 (71.8%)
	Yes	8,570 (28.2%)

For peer review only - http://bmjopen.bmj.com/site/about/guidelines.xhtml

Comparability of aggregate ethnicity

Among pupils who had usable ethnicity data from both the NPD and CRIS (n=20,991), the two data sources agreed on 87.0% of categorisations, Cohen's kappa=0.79 (95% CI=0.78 to 0.79), indicating strong overall agreement.

Cross-tabulations between ethnicity data in the two data sources can be found in Table 2 (note that for the purposes of this table only, we have excluded pupils who were categorised as Chinese in either data source, in order to avoid disclosive cell counts). The majority of those categorised as White, Black, or Asian in the NPD were also categorised as such in CRIS (row percentages, Table 2), and vice versa (column percentages, Table 2). However, only 50.9% of those in the NPD's Mixed ethnic group were also classified as such in CRIS. Additionally, of those grouped as Other in CRIS, only 26.7% were also grouped as Other in the NPD; the same proportion were instead grouped as White (26.7%), and still more were grouped as Mixed (27.0%). Further detail on the proportion of disaggregated CRIS ethnicities categorised as consistent major ethnic groups in the NPD is available in Table S5.

For peer review only

For peer review only - http://bmjopen.bmj.com/site/about/guidelines.xhtml

Table 2: Cross-tabulations of CRIS and NPD ethnicity where ethnicity was available from both sources.

NPD		CRIS White n=11,850	CRIS Black n=5,743	CRIS Asian n=675	CRIS Mixed n=1,840	CRIS Other n=808	CRIS Total n=20,916
**White n=11,507**	Row %	96.1	0.7	0.2	1.2	1.9	100.0
	Column %	93.3	1.3	3.3	7.6	26.7	55.0
**Black n=5,532**	Row %	2.7	90.4	0.6	4.3	1.9	100.0
	Column %	1.3	87.1	5.2	13.0	13.2	26.5
**Asian n=631**	Row %	6.8	2.5	80.8	1.7	8.1	100.0
	Column %	0.4	0.3	75.6	0.6	6.3	3.0
**Mixed n=2,806**	Row %	17.3	21.0	3.1	50.9	7.8	100.0
	Column %	4.1	10.2	12.7	77.6	27.0	13.4
**Other n=440**	Row %	27.3	13.4	5.0	5.2	49.1	100.0
	Column %	1.0	1.0	3.3	1.3	26.7	2.1
**Total n=20,916**	Row %	56.7	27.5	3.2	8.8	3.9	100.0
	Column %	100.0	100.0	100.0	100.0	100.0	100.0

Note: For the purposes of this table only, we have excluded pupils who were categorised as Chinese in either data source, in order to avoid disclosive cell counts.
Abbreviations: CRIS=Clinical Record Interactive Search, NPD=National Pupil Database.

Association with educational and clinical outcomes

The association between ethnicity, Year 11 attainment and neurodevelopmental disorder varied according to which data source was used to code ethnicity. Asian and Other ethnic groups were at significantly higher odds of achieving expected attainment thresholds in Year 11 than White ethnic groups when ethnicity was derived from the NPD, but not when ethnicity was derived from CRIS (Table S6, Figure 1). Also, compared to White ethnic groups, Mixed ethnic groups were at lower odds of neurodevelopmental disorder only when

For peer review only - http://bmjopen.bmj.com/site/about/guidelines.xhtml

derived from the NPD, and Chinese ethnic groups were at lower odds of neurodevelopmental disorder only when derived from CRIS (Table S7, Figure 2). Associations with missing ethnicity data also varied according to which data source was used.

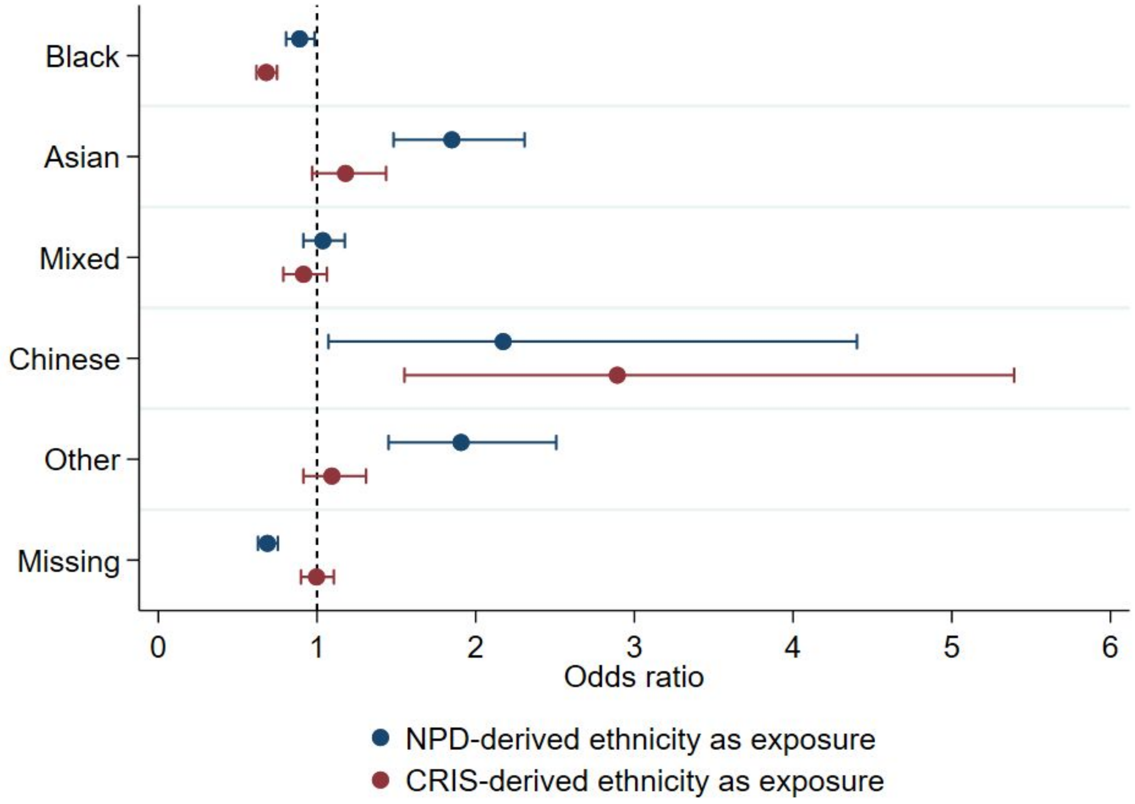

Figure 1: Plot of unadjusted logistic regression analyses between ethnicity and Year 11 expected attainment. White ethnicity is the reference category.

For peer review only - http://bmjopen.bmj.com/site/about/guidelines.xhtml

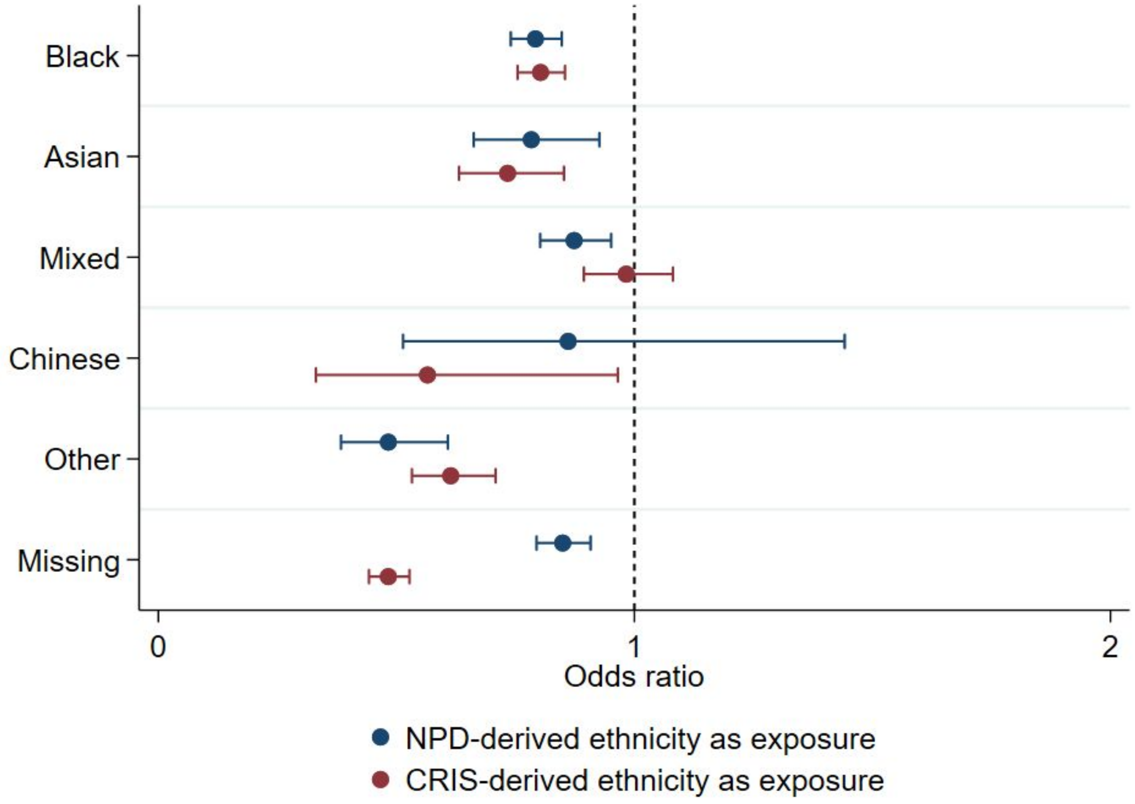

Figure 2: Plot of unadjusted logistic regression analyses between ethnicity and neurodevelopmental disorder diagnosis. White ethnicity is the reference category.

Discussion

These findings suggest that linkage between health and education data sources can provide an opportunity to increase available ethnicity data for studies using linked and routinely collected records. In this sample, the proportion with available and usable ethnicity data rose from 79.3% (from the NPD) and 87.0% (from CRIS) to 97.3% (from either the NPD or from CRIS). Ethnicity at an aggregate level mostly aligned between the two data sources, with the NPD and CRIS agreeing on 87.0% of classifications. However, as with previous studies, some divergence was observed, particularly in the Mixed and Other ethnic groups.[14]

Low quality ethnicity data is known to pose a challenge for investigating ethnic inequalities in public health.[5] A previous United Kingdom study found incomplete and

For peer review only - http://bmjopen.bmj.com/site/about/guidelines.xhtml

inconsistent ethnicity recordings in two health datasets,[14] and outputs accessed via the United Kingdom government's online data service corroborate that collection and reporting of ethnicity data within the public sector is not consistent.[24]

This study updates and extends previous findings by comparing two health and education datasets, thereby taking a cross-disciplinary perspective on this issue. Consistent with previous work, we found that linkage between these datasets increased availability of ethnicity data, but that the datasets did not consistently agree on ethnicity, particularly for Mixed and Other ethnic groups. The Other ethnic category is known to capture particularly heterogenous groups, such that non-alignment on this category is perhaps unsurprising. Indeed, the Other classification in each source may partly comprise individuals who do not feel that their ethnicity is otherwise captured by options offered by the data collection procedure. For example, when submitting ethnicity data for the school census, a range of ethnicity options are offered by the NPD which would ultimately be aggregated as White (such as Cornish, Italian, White Eastern European and White Western European). However, these options are not available in CRIS, such that in the clinical setting the patient, parent or recording clinician might instead select "any other group" as an alternative and be aggregated as Other. Disagreements between the data sources on the Mixed ethnic group might reflect previous United Kingdom research findings that individuals from Mixed ethnic groups are culturally positioned between White and minority ethnic groups, and how they self-identify may be particularly fluid and context-dependent.[25,26]

Therefore, some inconsistencies in ethnicity between multiple data sources are expected. Ethnicity is not a time-invariant characteristic, is highly context specific, and self-reported ethnic identity has been known to vary in the same individuals over the life course.[27] However, our findings suggest that this can result in substantial differences in estimated associations between ethnicity and important clinical and educational outcomes.

For peer review only - http://bmjopen.bmj.com/site/about/guidelines.xhtml

Our findings pose serious challenges for accurately identifying inequalities which need to be addressed. A recent report recommended the enforcement of guidelines to ensure that patient ethnicity is recorded, and recorded accurately based on self-report, during interactions with healthcare services. Doing so might resolve some of the observed discrepancies,[5] particularly as our study indicated large differences in observed associations for children with 'missing' ethnicity. However, it is important to note that both the coverage and quality of ethnicity recording in routinely collected data need to improve; solely enforcing more comprehensive completion of ethnicity fields could cause the quality and accuracy of these data to deteriorate further if not accompanied by updated guidance on recording ethnicity.[28]

Some of the issues we identified might also be resolved by data sources supplying ethnicity data to researchers at a more granular level to ensure consistency in how ethnicity is aggregated. Understandably, concerns around data anonymity sometimes preclude this, but in light of known inequalities at the level of more granular ethnicity and growing 'super-diversity' in the United Kingdom, aggregate ethnicity data remain insufficient.[29,30] Where aggregation is essential for maintaining anonymity, other methodological improvements might include standardisation across the public sector in how ethnicity is aggregated, or allowing researchers to specify how they want ethnicity to be aggregated when requesting data extracts.

Efforts to effectively capture ethnicity in research are ongoing. The Office for National Statistics conducted a public consultation and stakeholder survey to inform ethnic group data collection techniques in preparation for the 2021 Census. Their findings indicated that aggregated ethnicity categories (such as White, Black, Asian, Mixed and Other) do not sufficiently meet the requirements of many data users.[31] Our findings reinforce that not only is aggregate ethnicity data insufficient, but that it does not always align between large-scale

For peer review only - http://bmjopen.bmj.com/site/about/guidelines.xhtml

data sources. Moreover, choosing which data source to primarily code ethnicity from could have substantial implications for how associations between ethnicity, health and educational outcomes are interpreted, such that users of linked data should be cautious in combining and comparing ethnicity between different data sources.

We used broad terminology in reference to ethnicity, which is not desirable; however, as discussed, a more specific examination and discussion was precluded by only having access to aggregate ethnicity variables.[6] Additionally, the terminology follows Office for National Statistics standard approaches for aggregation which are commonly used by researchers and government analysts to inform United Kingdom policy. This is helpful for generalisability.

We only used one assessment of ethnicity per individual and per data source, and we were unable to ascertain exactly when ethnicity was ascribed. Therefore within-person changes in reported ethnicity over time could not be assessed.

We did not adjust the regression models for key covariates such as socioeconomic status, gender and age. It should be emphasised that our aim was not to provide a precise estimate of the association between ethnicity and attainment or neurodevelopmental disorder, but to ascertain whether variable findings can arise when measuring ethnicity from different data sources. Moreover, gender, socioeconomic status, and age were also recorded inconsistently between the datasets, so including them would raise further questions as to which data source to rely on for their classification: this is beyond the scope of this study's focus on discrepancies seen in ethnicity. However, whether these characteristics in turn predict ethnicity misclassification would be an area for future research. We also did not consider other important characteristics such as religion, first language and country of birth – future work could consider these characteristics to investigate whether linkage might inform inequalities research from an intersectional perspective.[32]

For peer review only - http://bmjopen.bmj.com/site/about/guidelines.xhtml

Conclusion

Data linkage minimises missingness in ethnicity data, and ethnicity categorisations between CRIS and the NPD were mostly consistent. However, choosing which data source to primarily code ethnicity from can impact findings relating to ethnicity, mental health, and education.

Funding statement

This paper represents independent research funded by the National Institute for Health Research (NIHR) Biomedical Research Centre at South London and Maudsley NHS Foundation Trust and King's College London (NIHR-INF-0690). The views expressed are those of the authors and not necessarily those of the NIHR, the Department of Health and Social Care or King's College London.

For peer review only

Competing interests

AW is supported by ADR UK (Administrative Data Research UK), an Economic and Social Research Council (ESRC) investment (part of UK Research and Innovation) (ES/W002531/1). RS is part-funded by: i) the National Institute for Health Research (NIHR) Biomedical Research Centre at the South London and Maudsley NHS Foundation Trust and King's College London; ii) the National Institute for Health Research (NIHR) Applied Research Collaboration South London (NIHR ARC South London) at King's College Hospital NHS Foundation Trust; iii) the DATAMIND HDR UK Mental Health Data Hub (MRC grant MR/W014386). RS has received research support in the last 3 years from Janssen, GSK and Takeda. JD is supported by NIHR Clinician Science Fellowship award (CS-2018-18-ST2-014) and has received support from a Medical Research Council (MRC) Clinical Research Training Fellowship (MR/L017105/1) and Psychiatry Research Trust Peggy Pollak Research Fellowship in Developmental Psychiatry. JDM is supported by the

For peer review only - http://bmjopen.bmj.com/site/about/guidelines.xhtml

ESRC Centre for Society and Mental Health at King's College London (ESRC Reference: ES/S012567/1), grants from the ESRC (ES/S002715/1) and by the National Institute for Health Research (NIHR) Biomedical Research Centre at South London and Maudsley NHS Foundation Trust and King's College London and the National Institute for Health Research (NIHR) Applied Research Collaboration South London (NIHR ARC South London) at King's College Hospital NHS Foundation Trust. The views expressed are those of the authors and not necessarily those of the ESRC, NIHR, the Department of Health and Social Care or King's College London.

Author contributions

AW conceived and designed the study, performed data analysis, and wrote the manuscript. JD led the data linkage, conducted initial cleaning of NPD data, and provided critical comments on the manuscript. AJ performed data extraction. JDM, TF, and RS consulted on study design and interpretation, and provided critical comments on the manuscript. All authors read and approved the final manuscript. At the time of undertaking this study, JD and AW had full access to the dataset from which the study population was derived.

Data sharing statement

The data that support the findings of this study are available from the Department for Education and South London and Maudsley NHS Foundation Trust, but restrictions apply to the availability of these data, and so they are not publicly available. For the purposes of open access, the author has applied a Creative Commons Attribution (CC BY) licence to any Accepted Author Manuscript version arising from this submission.

For peer review only - http://bmjopen.bmj.com/site/about/guidelines.xhtml

References

1. Department for Education. *Key stage 4 performance 2019 (revised)*. 2020. Accessed 14 October 2021. https://www.gov.uk/government/statistics/key-stage-4-performance-2019-revised
2. Wickersham A, Dickson H, Jones R, et al. Educational attainment trajectories among children and adolescents with depression, and the role of sociodemographic characteristics: longitudinal data-linkage study. *The British Journal of Psychiatry*. 2020;218(3):151-157. doi:10.1192/bjp.2020.160
3. Wickersham A, Ford T, Stewart R, Downs J. Estimating the impact of child and early adolescent depression on subsequent educational attainment: secondary analysis of an existing data linkage. *Epidemiology and Psychiatric Sciences*. 2021;30:e76. e76. doi:10.1017/S2045796021000603
4. Tromans S, Chester V, Gemegah E, et al. Autism identification across ethnic groups: a narrative review. *Advances in Autism*. 2020;7(3):241-255. doi:10.1108/AIA-03-2020-0017
5. Kapadia D, Zhang J, Salway S, et al. *Ethnic Inequalities in Healthcare: A Rapid Evidence Review*. 2022.
6. Bhopal R. Glossary of terms relating to ethnicity and race: for reflection and debate. *Journal of Epidemiology & Community Health*. 2004;58(6):441-445. doi:10.1136/jech.2003.013466
7. Hickman MJ, Ryan L. The "Irish question": marginalizations at the nexus of sociology of migration and ethnic and racial studies in Britain. *Ethnic and Racial Studies*. 2020;43(16):96-114. doi:10.1080/01419870.2020.1722194
8. Senior PA, Bhopal R. Ethnicity as a variable in epidemiological research. *BMJ*. 1994;309:327. doi:10.1136/bmj.309.6950.327
9. Boehmer U, Kressin NR, Berlowitz DR, Christiansen CL, Kazis LE, Jones JA. Self-reported vs administrative race/ethnicity data and study results. *American Journal of Public Health*. 2002;92(9):1471-1472. doi:10.2105/AJPH.92.9.1471
10. López MM, Bevans M, Wehrlen L, Yang L, Wallen G. Discrepancies in race and ethnicity documentation: a potential barrier in identifying racial and ethnic disparities. *Journal of Racial and Ethnic Health Disparities*. 2017;4:812-818. doi:10.1007/s40615-016-0283-3
11. McAlpine DD, Beebe TJ, Davern M, Call KT. Agreement between self-reported and administrative race and ethnicity data among Medicaid enrollees in Minnesota. *Health services research*. 2007;42(6p2):2373-2388. doi:10.1111/j.1475-6773.2007.00771.x
12. Saunders CL, Abel GA, El Turabi A, Ahmed F, Lyratzopoulos G. Accuracy of routinely recorded ethnic group information compared with self-reported ethnicity: evidence from the English Cancer Patient Experience survey. *BMJ Open*. 2013;3:e002882. doi:10.1136/bmjopen-2013-002882
13. Scobie S, Spencer J, Raleigh V. Ethnicity coding in English health service datasets. Accessed 11 November 2021, https://www.nuffieldtrust.org.uk/research/ethnicity-coding-in-english-health-service-datasets
14. Mathur R, Bhaskaran K, Chaturvedi N, et al. Completeness and usability of ethnicity data in UK-based primary care and hospital databases. *Journal of Public Health*. 2013;36(4):684-692. doi:10.1093/pubmed/fdt116
15. Office for National Statistics. Data from: 2011 census: ethnic group (detailed), local authorities in England and Wales. 2013. *2011 Census: Key Statistics and Quick Statistics for Wards and Output Areas in England and Wales*.
16. Downs J, Gilbert R, Hayes RD, Hotopf M, Ford T. Linking health and education data to plan and evaluate services for children. *Archives of Disease in Childhood*. 2017;102(7):599-602. doi:10.1136/archdischild-2016-311656
17. Downs J, Ford T, Stewart R, et al. An approach to linking education, social care and electronic health records for children and young people in South London: a linkage study of child and adolescent mental health service data. *BMJ Open*. 2019;9(1):e024355. doi:10.1136/bmjopen-2018-024355
18. Jay MA, Mc Grath-Lone L, Gilbert R. Data resource: the National Pupil Database (NPD). *International Journal of Population Data Science*. 2019;4(1)doi:10.23889/ijpds.v4i1.1101

For peer review only - http://bmjopen.bmj.com/site/about/guidelines.xhtml

19. Office for National Statistics. Ethnic group, national identity and religion. Accessed 07 September 2020, https://www.ons.gov.uk/methodology/classificationsandstandards/measuringequality/ethnicgroupnationalidentityandreligion
20. Cohen J. A coefficient of agreement for nominal scales. *Educational and Psychological Measurement*. 1960;20(1):37-46. doi:10.1177/001316446002000104
21. McHugh ML. Interrater reliability: the kappa statistic. *Biochemia Medica*. 2012;22(3):276-282. doi:10.11613/BM.2012.031
22. Thapar A, Cooper M, Rutter M. Neurodevelopmental disorders. *The Lancet Psychiatry*. 2017/04/01/ 2017;4(4):339-346. doi:10.1016/S2215-0366(16)30376-5
23. *Stata Statistical Software: Release 15*. StataCorp LLC; 2017.
24. Cabinet Office. The 2021 census of population and housing in England and Wales. https://www.gov.uk/government/publications/the-2021-census-of-population-and-housing-in-england-and-wales
25. Barrett M, Eade J, Cinnirella M, Garbin D. *New ethnicities among British Bangladeshi and mixed-heritage youth*. 2006. *The Leverhulme Trust*.
26. Panico L, Nazroo JY. The social and economic circumstances of mixed ethnicity children in the UK: findings from the Millennium Cohort Study. *Ethnic and Racial Studies*. 2011;34(9):1421-1444. doi:10.1080/01419870.2011.556745
27. Simpson L. *How have people's ethnic identities changed in England and Wales?* 2014. *The Dynamics of Diversity: evidence from the 2011 Census*. Accessed 14 October 2021. https://hummedia.manchester.ac.uk/institutes/code/briefingsupdated/how-have-people's-ethnic-identities-changed-in-england-and-wales.pdf
28. Raleigh V, Goldblatt P. Ethnicity coding in health records. Accessed 01 April 2022, https://www.kingsfund.org.uk/publications/ethnicity-coding-health-records
29. Strand S. Ethnicity, gender, social class and achievement gaps at age 16: intersectionality and 'getting it' for the white working class. *Research Papers in Education*. 2014;29(2):131-171. doi:10.1080/02671522.2013.767370
30. Vertovec S. Super-diversity and its implications. *Ethnic and Racial Studies*. 2007;30(6):1024-1054. doi:10.1080/01419870701599465
31. Office for National Statistics. 2021 census: ethnic group stakeholder follow-up survey – summary of responses. Accessed 07 September 2020, https://www.ons.gov.uk/census/censustransformationprogramme/questiondevelopment/ethnicgroupnationalidentityreligionandlanguage/2021censusethnicgroupstakeholderfollowupsurveysummaryofresponses
32. Gazard B, Frissa S, Nellums L, Hotopf M, Hatch SL. Challenges in researching migration status, health and health service use: an intersectional analysis of a South London community. *Ethnicity & Health*. 2015;20(6):564-593. doi:10.1080/13557858.2014.961410

For peer review only - http://bmjopen.bmj.com/site/about/guidelines.xhtml

Table S1: The RECORD statement – checklist of items, extended from the STROBE statement, that should be reported in observational studies using routinely collected health data. Items specific to cross-sectional/case-control studies have been removed.

	Item No.	STROBE items	Page	RECORD items	Page
**Title and abstract**					
	1	(a) Indicate the study's design with a commonly used term in the title or the abstract (b) Provide in the abstract an informative and balanced summary of what was done and what was found	2	RECORD 1.1: The type of data used should be specified in the title or abstract. When possible, the name of the databases used should be included.<br><br>RECORD 1.2: If applicable, the geographic region and timeframe within which the study took place should be reported in the title or abstract.<br><br>RECORD 1.3: If linkage between databases was conducted for the study, this should be clearly stated in the title or abstract.	2
**Introduction**					
Background rationale	2	Explain the scientific background and rationale for the investigation being reported	3-4	N/A	
Objectives	3	State specific objectives, including any prespecified hypotheses	3-4	N/A	
**Methods**					
Study Design	4	Present key elements of study design early in the paper	5	N/A	
Setting	5	Describe the setting, locations, and relevant dates, including periods of recruitment, exposure, follow-up, and data collection	5	N/A	
Participants	6	(a) Cohort study - Give the eligibility criteria, and the sources and methods of selection of participants. Describe methods of follow-up<br><br>(b) Cohort study - For matched studies, give matching criteria and number of exposed and unexposed	5	RECORD 6.1: The methods of study population selection (such as codes or algorithms used to identify subjects) should be listed in detail. If this is not possible, an explanation should be provided.<br><br>RECORD 6.2: Any validation studies of the codes or algorithms used to select the population should be referenced. If validation	5

For peer review only

	Item No.	STROBE items	Page	RECORD items	Page
				was conducted for this study and not published elsewhere, detailed methods and results should be provided.<br><br>RECORD 6.3: If the study involved linkage of databases, consider use of a flow diagram or other graphical display to demonstrate the data linkage process, including the number of individuals with linked data at each stage.	
Variables	7	Clearly define all outcomes, exposures, predictors, potential confounders, and effect modifiers. Give diagnostic criteria, if applicable.	6-7	RECORD 7.1: A complete list of codes and algorithms used to classify exposures, outcomes, confounders, and effect modifiers should be provided. If these cannot be reported, an explanation should be provided.	6-7
Data sources/ measurement	8	For each variable of interest, give sources of data and details of methods of assessment (measurement).<br>Describe comparability of assessment methods if there is more than one group	6-7	N/A	
Bias	9	Describe any efforts to address potential sources of bias	6-7	N/A	
Study size	10	Explain how the study size was arrived at	5	N/A	
Quantitative variables	11	Explain how quantitative variables were handled in the analyses. If applicable, describe which groupings were chosen, and why	N/A	N/A	
Statistical methods	12	(a) Describe all statistical methods, including those used to control for confounding<br>(b) Describe any methods used to examine subgroups and interactions<br>(c) Explain how missing data were addressed<br>(d) Cohort study - If applicable, explain how loss to follow-up was addressed<br>(e) Describe any sensitivity analyses	7	N/A	
Data access and cleaning methods		N/A		RECORD 12.1: Authors should describe the extent to which the investigators had access to the database population used to create the study population.	5, 17

For peer review only

	Item No.	STROBE items	Page	RECORD items	Page
				RECORD 12.2: Authors should provide information on the data cleaning methods used in the study.	
Linkage		N/A		RECORD 12.3: State whether the study included person-level, institutional-level, or other data linkage across two or more databases. The methods of linkage and methods of linkage quality evaluation should be provided.	5
**Results**					
Participants	13	(a) Report the numbers of individuals at each stage of the study (e.g., numbers potentially eligible, examined for eligibility, confirmed eligible, included in the study, completing follow-up, and analysed)<br>(b) Give reasons for non-participation at each stage.<br>(c) Consider use of a flow diagram	Supp leme nt	RECORD 13.1: Describe in detail the selection of the persons included in the study (i.e., study population selection) including filtering based on data quality, data availability and linkage. The selection of included persons can be described in the text and/or by means of the study flow diagram.	5
Descriptive data	14	(a) Give characteristics of study participants (e.g., demographic, clinical, social) and information on exposures and potential confounders<br>(b) Indicate the number of participants with missing data for each variable of interest<br>(c) Cohort study - summarise follow-up time (e.g., average and total amount)	8	N/A	
Outcome data	15	Cohort study - Report numbers of outcome events or summary measures over time	8	N/A	
Main results	16	(a) Give unadjusted estimates and, if applicable, confounder-adjusted estimates and their precision (e.g., 95% confidence interval). Make clear which confounders were adjusted for and why they were included<br>(b) Report category boundaries when continuous variables were categorized<br>(c) If relevant, consider translating estimates of relative risk into absolute risk for a meaningful time period	10-12	N/A	

For peer review only

	Item No.	STROBE items	Page	RECORD items	Page
Other analyses	17	Report other analyses done—e.g., analyses of subgroups and interactions, and sensitivity analyses	9-10	N/A	
**Discussion**					
Key results	18	Summarise key results with reference to study objectives	12-13	N/A	
Limitations	19	Discuss limitations of the study, taking into account sources of potential bias or imprecision. Discuss both direction and magnitude of any potential bias	15	RECORD 19.1: Discuss the implications of using data that were not created or collected to answer the specific research question(s). Include discussion of misclassification bias, unmeasured confounding, missing data, and changing eligibility over time, as they pertain to the study being reported.	15
Interpretation	20	Give a cautious overall interpretation of results considering objectives, limitations, multiplicity of analyses, results from similar studies, and other relevant evidence	12-16	N/A	
Generalisability	21	Discuss the generalisability (external validity) of the study results	15	N/A	
**Other Information**					
Funding	22	Give the source of funding and the role of the funders for the present study and, if applicable, for the original study on which the present article is based	16	N/A	
Accessibility of protocol, raw data, and programming code		N/A		RECORD 22.1: Authors should provide information on how to access any supplemental information such as the study protocol, raw data, or programming code.	17

*Reference: Benchimol, E. I., Smeeth, L., Guttmann, A., Harron, K., Moher, D., Petersen, I., ... & RECORD Working Committee. (2015). The REporting of studies Conducted using Observational Routinely-collected health Data (RECORD) statement. PLoS Med, 12(10), e1001885.

*Checklist is protected under Creative Commons Attribution (CC BY) license.

For peer review only

Figure S1: Study flow diagram.

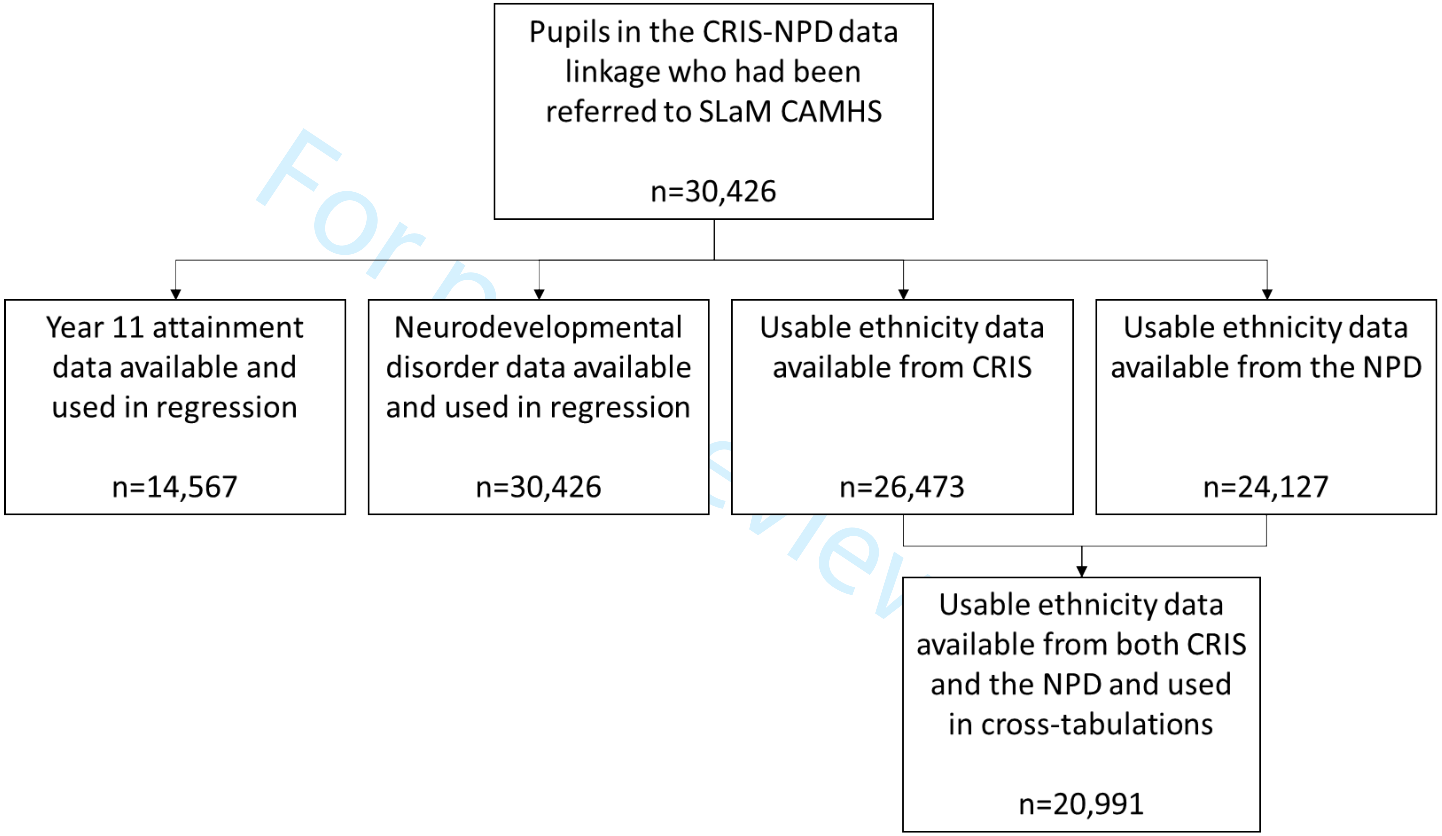

Table S2: Ethnic groups in the NPD.

Approved extended categories	Minor ethnic group	Major ethnic group
White – British	White - British	White
White – Cornish [a]		
White – English		
White – Scottish		
White – Welsh		
Other White British		
White – Irish	White	
Traveller of Irish heritage		
Any other White background	Any other White background	
Albanian		
Bosnian-Herzegovinian		
Croatian		
Greek/Greek Cypriot		
Greek		
Greek Cypriot		
Italian [a]		
Kosovan		
Portuguese		
Serbian		
Turkish/Turkish Cypriot		
Turkish		
Turkish Cypriot		
White European [a]		
White Eastern European [a]		
White Western European [a]		
White other		
Gypsy/Roma	Gypsy/Roma	
Gypsy		
Roma		
Other Gypsy/Roma		
White and Black Caribbean	White and Black Caribbean	Mixed/Dual background
White and Black African	White and Black African	
White and Asian	White and Asian	
White and Pakistani [a]		
White and Indian [a]		
White and any other Asian background		
Any other Mixed background	Any other Mixed background	
Asian and any other ethnic group [a]		
Asian and Black		
Asian and Chinese		
Black and any other ethnic group [a]		
Black and Chinese		

For peer review only

Approved extended categories	Minor ethnic group	Major ethnic group
Chinese and any other ethnic group [a]		
White and any other ethnic group [a]		
White and Chinese		
Other Mixed background		
Indian	Indian	Asian or Asian British
Pakistani	Pakistani	
Mirpuri Pakistani [a]		
Kashmiri Pakistani [a]		
Other Pakistani		
Bangladeshi	Bangladeshi	
Any other Asian background	Any other Asian background	
African Asian [a]		
Kashmiri other [a]		
Nepali [a]		
Sri Lankan Sinhalese		
Sri Lankan Tamil		
Sri Lankan other		
Other Asian		
Black Caribbean	Black Caribbean	Black or Black British
Black - African	Black - African	
Black - Angolan		
Black - Congolese [a]		
Black – Ghanaian		
Black – Nigerian		
Black - Sierra Leonean [a]		
Black – Somali		
Black – Sudanese		
Other Black African		
Any other Black background	Any other Black background	
Black European [a]		
Black North American [a]		
Other Black		
Chinese	Chinese	Chinese
Hong Kong Chinese [a]		
Malaysian Chinese		
Singaporean Chinese [a]		
Taiwanese [a]		
Other Chinese		
Any other ethnic group	Any other ethnic group	Any other ethnic group
Afghan [a]		
Arab other		
Egyptian [a]		
Filipino		
Iranian		

For peer review only

Approved extended categories	Minor ethnic group	Major ethnic group
Iraqi		
Japanese		
Korean [a]		
Kurdish		
Latin/South/Central American [a]		
Lebanese [a]		
Libyan [a]		
Malay		
Moroccan [a]		
Polynesian [a]		
Thai [a]		
Vietnamese		
Yemeni		
Other ethnic group		
Refused	Refused	Refused
Information not yet obtained	Information not yet obtained	Information not yet obtained

a. Specific ethnicities not included in the SLaM electronic health record ethnic categories list (CRIS, Supplement 4).
Note: Contains public sector information licensed under the Open Government Licence v3.0.

For peer review only

Table S3: Ethnic groups in CRIS.

SLaM electronic health record ethnic categories	16+1 ethnic categories supplied from CRIS
British (A)	British (A)
English (CA)	
Scottish (CB)	
Welsh (CC)	
Irish (B)	Irish (B)
Irish Traveller (CL)	
Albanian (CS)	Any other White background €
All former USSR Republics (CQ) [a]	
Bosnian (CT)	
Croatian (CU)	
Cypriot (part not stated) (CE) [a]	
Greek (CF)	
Greek Cypriot (CG)	
Gypsy/Romany (CN)	
Kosovan (CR)	
Kurdish (C5)	
Other former Yugoslavia (CW) [a]	
Other White Unspecified (C3)	
Other White/Mixed European (CY) [a]	
Portuguese (C4)	
Serbian (CV)	
Traveller (CM) [a]	
Turkish (CH)	
Turkish Cypriot (CJ)	
White and Black Caribbean (D)	White and Black Caribbean (D)
White and Black African €	White and Black African €
White and Asian (F)	White and Asian (F)
Asian and Chinese (GE)	Any other Mixed background (G)
Black and Asian (GA)	
Black and Chinese (GB)	
Black and White (GC) [a]	
Chinese and White (GD)	
Indian/British Indian (H)	Indian (H)
Pakistani/British Pakistani (J)	Pakistani (J)
Bangladeshi/British Bangladeshi (K)	Bangladeshi (K)
British Asian (LH) [a]	Any other Asian background (L)
Caribbean Asian (LJ) [a]	
East African Asian (LD) [a]	
Mixed Asian (LA) [a]	
Other Asian Unspecified (LK)	
Sinhalese (LG)	
Sri Lankan (LE)	
Tamil (LF)	

For peer review only

SLaM electronic health record ethnic categories	16+1 ethnic categories supplied from CRIS
Caribbean (M)	Caribbean (M)
Algerian (PP) [a]<br>Angolan (PJ)<br>Eritrean (PK) [a]<br>Ethiopian (PL) [a]<br>Ghanaian (PM)<br>Nigerian (PC)<br>Somali (PA)<br>Sudanese (PH)<br>Ugandan (PQ) [a]<br>Other African (N)	African (N)
Mixed Black (PB) [a]<br>Black British (PD) [a]<br>Other Black Unspecified (PE)	Any other Black background (P)
Chinese (R)	Chinese (R)
Any Other Group (SE)<br>Arab (SG)<br>Columbian (SK) [a]<br>Ecuadorian (SL) [a]<br>Filipino (SC)<br>Iranian (SH)<br>Iraqi (SJ)<br>Japanese (SB)<br>Malaysian (SD)<br>Middle Eastern (SF) [a]<br>Other Latin American (SM)<br>Vietnamese (SA)	Any other ethnic group (S)
Not Stated (Z)	Not Stated (Z)

a. Specific ethnicities not included in the 'approved extended ethnic categories' list (NPD, Supplement 3).

For peer review only

Table S4: Aggregating CRIS ethnicity to assist comparison with the NPD.

16+1 ethnic categories supplied from CRIS	Aggregate category
British (A)<br>Irish (B)<br>Any other White background (C)	White
White and Black Caribbean (D)<br>White and Black African (E)<br>White and Asian (F)<br>Any other Mixed background (G)	Mixed
Indian (H)<br>Pakistani (J)<br>Bangladeshi (K)<br>Any other Asian background (L)	Asian
Caribbean (M)<br>African (N)<br>Any other Black background (P)	Black
Chinese (R)	Chinese
Any other ethnic group (S)	Other
Not Stated (Z)	Unknown

For peer review only

Table S5: Proportion of disaggregated CRIS ethnicities categorised as consistent major ethnic groups in the NPD, among individuals where ethnicity was available from both sources (n=20,991).

		NPD					
		**White n=11,507**	**Black n=5,532**	**Asian n=632**	**Chinese n=68**	**Mixed n=2,810**	**Other n=442**
CRIS	**British n=10,634**	10,056 (94.6%)					
	**Irish n=129**	121 (93.8%)					
	**Any other White background n=1,090**	877 (80.5%)					
	**African n=1,216**		1,114 (91.6%)				
	**Caribbean n=1,207**		1,089 (90.2%)				
	**Any other Black background n=3,320**		2,800 (84.3%)				
	**Indian n=170**			142 (83.5%)			
	**Pakistani n=129**			116 (89.9%)			
	**Bangladeshi n=111**			105 (94.6%)			
	**Any other Asian background n=268**			147 (54.9%)			
	**Chinese n=58**				51 (87.9%)		
	**White and Asian n=134**					104 (77.6%)	
	**White and Black African n=238**					179 (75.2%)	
	**White and Black Caribbean n=1,102**					873 (79.2%)	
	**Any other Mixed background n=372**					272 (73.1%)	
	**Any other ethnic group n=813**						216 (26.6%)

Note: Row percentages provided. Off-diagonal proportions have been suppressed to avoid disclosive cell counts.

For peer review only

Table S6: Unadjusted logistic regression analyses between ethnicity derived from either NPD or CRIS (exposure) and whether the Year 11 expected attainment threshold was achieved (outcome; 'no' is the reference group)

	NPD-derived ethnicity as exposure				CRIS-derived ethnicity as exposure			
	No (n, %)	Yes (n, %)	OR (95% CI)	p	No (n, %)	Yes (n, %)	OR (95% CI)	p
**White**	4,485 (67.1%)	2,202 (32.9%)	Reference	-	5,041 (67.2%)	2,461 (32.8%)	Reference	-
**Black**	1,691 (69.6%)	739 (30.4%)	0.89 (0.81 to 0.98)	0.023	2,293 (75.1%)	761 (24.9%)	0.68 (0.62 to 0.75)	<0.001
**Asian**	174 (52.4%)	158 (47.6%)	1.85 (1.48 to 2.31)	<0.001	290 (63.5%)	167 (36.5%)	1.18 (0.97 to 1.44)	0.099
**Chinese**	15 (51.6%)	16 (51.6%)	2.17 (1.07 to 4.40)	0.031	17 (41.5%)	24 (58.5%)	2.89 (1.55 to 5.39)	0.001
**Mixed**	861 (66.3%)	438 (33.7%)	1.04 (0.91 to 1.18)	0.580	618 (69.1%)	276 (30.9%)	0.91 (0.79 to 1.06)	0.244
**Other**	110 (51.6%)	103 (48.4%)	1.91 (1.45 to 2.51)	<0.001	369 (65.2%)	197 (34.8%)	1.09 (0.91 to 1.31)	0.329
**Missing**	2,673 (74.8%)	902 (25.2%)	0.69 (0.63 to 0.75)	<0.001	1,381 (67.3%)	672 (32.7%)	1.00 (0.90 to 1.11)	0.951

Note: n=14,567. The n=15,859 not included in these analyses were missing data on Year 11 attainment.
Abbreviations: CRIS=Clinical Record Interactive Search, NPD=National Pupil Database.

For peer review only

Table S7: Unadjusted logistic regression analyses between ethnicity derived from either NPD or CRIS (exposure) and neurodevelopmental disorder diagnosis (outcome; 'no' is the reference group)

	NPD-derived ethnicity as exposure				CRIS-derived ethnicity as exposure			
	**No (%)**	**Yes (%)**	**OR (95% CI)**	**p**	**No (%)**	**Yes (%)**	**OR (95% CI)**	**p**
**White**	9,218 (69.5%)	4,041 (30.5%)	Reference	-	9,963 (68.4%)	4,609 (31.6%)	Reference	-
**Black**	4,660 (74.2%)	1,618 (25.8%)	0.79 (0.74 to 0.85)	<0.001	5,396 (72.9%)	2,004 (27.1%)	0.80 (0.75 to 0.85)	<0.001
**Asian**	562 (74.4%)	193 (25.6%)	0.78 (0.66 to 0.93)	0.004	716 (74.7%)	243 (25.3%)	0.73 (0.63 to 0.85)	<0.001
**Chinese**	53 (72.6%)	20 (27.4%)	0.86 (0.51 to 1.44)	0.569	65 (79.3%)	17 (20.7%)	0.57 (0.33 to 0.97)	0.037
**Mixed**	2,348 (72.3%)	899 (27.7%)	0.87 (0.80 to 0.95)	0.002	1,577 (68.7%)	717 (31.3%)	0.98 (0.89 to 1.08)	0.720
**Other**	425 (82.5%)	90 (17.5%)	0.48 (0.38 to 0.61)	<0.001	908 (77.9%)	258 (22.1%)	0.61 (0.53 to 0.71)	<0.001
**Missing**	4,590 (72.9%)	1,709 (27.1%)	0.85 (0.79 to 0.91)	<0.001	3,231 (81.7%)	722 (18.3%)	0.48 (0.44 to 0.53)	<0.001

Note: n=30,426.
Abbreviations: CRIS=Clinical Record Interactive Search, NPD=National Pupil Database.

For peer review only

BMJ Open

The impact of inconsistent ethnicity recordings on estimates of inequality in child health and education data: A data linkage study of Child and Adolescent Mental Health services in South London

Journal:	*BMJ Open*
Manuscript ID	bmjopen-2023-078788.R1
Article Type:	Original research
Date Submitted by the Author:	27-Nov-2023
Complete List of Authors:	Wickersham, Alice; King's College London, CAMHS Digital Lab, Department of Child and Adolescent Psychiatry<br>Das-Munshi, Jayati; King's College London, Psychological Medicine<br>Ford, Tamsin; University of Cambridge, Psychiatry<br>Jewell, Amelia; South London and Maudsley NHS Foundation Trust, Maudsley Biomedical Research Centre<br>Stewart, Robert; King's College London, Department of Psychological Medicine<br>Downs, Johnny; King's College London, CAMHS Digital Lab, Department of Child and Adolescent Psychiatry
**Primary Subject Heading**:	Epidemiology
Secondary Subject Heading:	Public health, Mental health, Research methods
Keywords:	MENTAL HEALTH, Health Equity, EDUCATION & TRAINING (see Medical Education & Training), EPIDEMIOLOGIC STUDIES, Child & adolescent psychiatry < PSYCHIATRY

SCHOLARONE™
Manuscripts

I, the Submitting Author has the right to grant and does grant on behalf of all authors of the Work (as defined in the below author licence), an exclusive licence and/or a non-exclusive licence for contributions from authors who are: i) UK Crown employees; ii) where BMJ has agreed a CC-BY licence shall apply, and/or iii) in accordance with the terms applicable for US Federal Government officers or employees acting as part of their official duties; on a worldwide, perpetual, irrevocable, royalty-free basis to BMJ Publishing Group Ltd ("BMJ") its licensees and where the relevant Journal is co-owned by BMJ to the co-owners of the Journal, to publish the Work in this journal and any other BMJ products and to exploit all rights, as set out in our licence.

The Submitting Author accepts and understands that any supply made under these terms is made by BMJ to the Submitting Author unless you are acting as an employee on behalf of your employer or a postgraduate student of an affiliated institution which is paying any applicable article publishing charge ("APC") for Open Access articles. Where the Submitting Author wishes to make the Work available on an Open Access basis (and intends to pay the relevant APC), the terms of reuse of such Open Access shall be governed by a Creative Commons licence – details of these licences and which Creative Commons licence will apply to this Work are set out in our licence referred to above.

Other than as permitted in any relevant BMJ Author's Self Archiving Policies, I confirm this Work has not been accepted for publication elsewhere, is not being considered for publication elsewhere and does not duplicate material already published. I confirm all authors consent to publication of this Work and authorise the granting of this licence.

For peer review only

The impact of inconsistent ethnicity recordings on estimates of inequality in child health and education data: A data linkage study of Child and Adolescent Mental Health services in South London

Alice Wickersham [a*], Jayati Das-Munshi [b], Tamsin Ford [c], Amelia Jewell [d], Robert Stewart [b] and Johnny Downs [a]

[a] CAMHS Digital Lab, Department of Child and Adolescent Psychiatry, King's College London, London, United Kingdom; [b] Department of Psychological Medicine, King's College London, London, United Kingdom; [c] Department of Psychiatry, University of Cambridge, Cambridge, United Kingdom; [d] South London and Maudsley NHS Foundation Trust, London, United Kingdom.

* Corresponding author. CAMHS Digital Lab, Department of Child and Adolescent Psychiatry, Institute of Psychiatry, Psychology and Neuroscience, King's College London, 16 De Crespigny Park, London, SE5 8AF

alice.wickersham@kcl.ac.uk

ORCID: 0000-0002-7402-7690

Word count (excludes the title page, abstract, tables, acknowledgements, contributions and references): 3250

For peer review only - http://bmjopen.bmj.com/site/about/guidelines.xhtml

Abstract

Objectives: Ethnicity data are critical for identifying inequalities, but previous studies suggest ethnicity is not consistently recorded between different administrative datasets. With researchers increasingly leveraging cross-domain data linkages, we investigated the completeness and consistency of ethnicity data in two linked health and education datasets.

Design: Cohort study.

Setting: South London and Maudsley NHS Foundation Trust de-identified electronic health records, accessed via Clinical Record Interactive Search (CRIS), and the National Pupil Database (NPD) (2007-2013).

Participants: n=30,426 children and adolescents referred to local Child and Adolescent Mental Health Services.

Primary and secondary outcome measures: Ethnicity data were compared between CRIS and the NPD. Associations between ethnicity as recorded from each source and key educational and clinical outcomes were explored with logistic regression.

Results: Ethnicity data were available for 79.3% from the NPD, 87.0% from CRIS, 97.3% from either source and 69.0% from both sources. Among those who had ethnicity data from both, the two data sources agreed on 87.0% of aggregate ethnicity categorisations overall, but with high levels of disagreement in Mixed and Other ethnic groups. Strengths of associations between ethnicity, educational attainment and neurodevelopmental disorder varied according to which data source was used to code ethnicity. For example, as compared to White pupils, a significantly higher proportion of Asian pupils achieved expected educational attainment thresholds only if ethnicity was coded from the NPD (RR=1.46, 95% CI=1.29 to 1.64), not if ethnicity was coded from CRIS (RR=1.11, 0.98 to 1.26).

Conclusions: Data linkage has the potential to minimise missing ethnicity data, and overlap in ethnicity categorisations between CRIS and the NPD was generally high. However, choosing which data source to primarily code ethnicity from can have implications for analyses of ethnicity, mental health, and educational outcomes. Users of linked data should exercise caution in combining and comparing ethnicity between different data sources.

Key words: Ethnicity, Mental Health, Education, Data Linkage, Administrative Data

For peer review only - http://bmjopen.bmj.com/site/about/guidelines.xhtml

Article summary: Strengths and limitations of this study

- We leveraged a data linkage between routinely-collected health and education datasets for a large cohort of children and adolescents referred to mental health services in South London.
- We conducted a comprehensive analysis of the availability and consistency of ethnicity data between these health and education datasets.
- Only one assessment of ethnicity was available from each data source and for each individual, precluding analysis of within-person changes in identity over time.
- Aggregate ethnicity data were primarily used for analysis.

Introduction

Ethnicity data are critical for highlighting inequalities in the population and informing public health and educational policies. For example, some minority ethnic groups are thought to perform less well in school assessments (1-3), and to be underdiagnosed with neurodevelopmental disorders (4). A recent review reported significant ethnic inequalities in access, experiences and outcomes of healthcare in the United Kingdom, but also highlighted that very few national datasets have sufficiently high quality ethnicity data to robustly investigate these inequalities (5). Incomplete and inconsistent ethnicity data in administrative datasets could result in variable estimates for important clinical and educational outcomes, compromising our ability to identify and address these inequalities.

Ethnicity is a fluid construct which should be self-ascribed and is socially constructed, capturing shared characteristics such as geography, ancestry, culture and language (6). Epidemiological studies using routinely collected data often rely on ethnicity variables which have been aggregated into several top-level categories. This is due to small sample sizes in more granular ethnic groups, associated concerns with statistical power, and the risk of inadvertent de-anonymisation which arises when sharing detailed ethnicity data. However,

For peer review only
For peer review only - http://bmjopen.bmj.com/site/about/guidelines.xhtml

aggregating ethnicity data results in problematic within-group heterogeneity, and risks specific ethnic groups being overlooked in research (6-8). Not only do these aggregate classifications fail to meaningfully capture homogenous groups, but they also do not necessarily align across different data sources. Previous research from the United States of America, Australia, and the United Kingdom has repeatedly raised concerns over the completeness and consistency of ethnicity variables in routinely collected health and other administrative data, particularly in comparison to self-reported survey data (9-14).

A recent United Kingdom report on Hospital Episode Statistics, the Emergency Care Data Set, and the Community Services Dataset found that ethnicity recordings in these datasets were often incomplete, and that for minority ethnic groups in particular, ethnicity codes were not used consistently for the same individuals over time (15). Another United Kingdom study compared two linked administrative health datasets from primary care and hospital settings, and found that combining the data sources increased the completeness of ethnicity data (16). While agreement between these sources was generally high, with 85% of patients having the same ethnicity recorded in both primary care and hospital data, agreement was much weaker for South Asian and Other ethnic groups.

We sought to explore this issue in linked administrative data from health and education domains for a cohort of pupils who had been referred to Child and Adolescent Mental Health Services (CAMHS) in South London. With cross-disciplinary data linkages increasingly being adopted for epidemiological research in this age group (17), an understanding of the completeness and consistency of ethnicity from two such data sources would be beneficial. This will help us to understand whether and how estimates of ethnic inequalities in health and education services might be affected by any measurement issues specific to these datasets and to populations of children and adolescents accessing mental health services.

For peer review only - http://bmjopen.bmj.com/site/about/guidelines.xhtml

The aims of this exploratory analysis were therefore to (1) summarise the completeness of ethnicity data in the linked health and education records; (2) summarise the alignment of aggregate ethnicity between the two data sources; and (3) investigate whether key educational and clinical outcomes vary according to which data source is used to code ethnicity.

Materials and methods

Data

We used an existing data linkage between de-identified electronic health records from South London and Maudsley NHS Foundation Trust (SLaM), and educational records from the Department for Education. SLaM serves the boroughs of Croydon, Lambeth, Lewisham and Southwark, and provides some national services. The SLaM catchment area is ethnically diverse, and has a lower proportion of individuals from White ethnic backgrounds compared to London and England as a whole (18), SLaM makes de-identified electronic health records available for research via the Clinical Record Interactive Search (CRIS) system. For children and adolescents (aged 4 to 18 years) referred to SLaM Child and Adolescent Mental Health Services (CAMHS) between 2007 and 2013, individual-level data linkage was undertaken between CRIS and the National Pupil Database (NPD) (19, 20). This analysis focused on pupils in the CRIS-NPD data linkage who had been referred to SLaM CAMHS, either from within the SLaM catchment area or from out-of-area (n=30,426). Reporting follows RECORD guidelines (Table S1 and Figure S1).

The CRIS data resource, including the linked data used in this manuscript, has received research ethics approval for secondary analyses (Oxford REC C, reference 18/SC/0372). The CRIS Oversight Committee ensures that research conducted using the

For peer review only - http://bmjopen.bmj.com/site/about/guidelines.xhtml

For peer review only

health records are ethical and legal, and service users can opt-out of their CRIS data being used for research.

Patient and public involvement statement

The data linkage used in this study was carried out in consultation with several patient and caregiver groups at the National Institute for Health Research Biomedical Research Centre, South London and Maudsley National Health Service Foundation Trust and King's College London. This has been described elsewhere (19, 20).

Ethnicity – NPD

In the NPD, pupil ethnicity is collected as part of the school census undertaken by England's state schools. It is either self-ascribed or parent-ascribed, although historically this information could also be ascribed by the school (21). Due to concerns over sensitivity and potential disclosure, ethnicity extracts from the NPD are sometimes supplied to researchers as what is termed 'major ethnic groups' only (Table S2). Major ethnic groups are similar to the aggregated groups used by the Office for National Statistics; however, Chinese is provided as a separate group rather than aggregated under Asian/Asian British (White, Black, Asian, Chinese, Mixed, Other, and Unknown) (22).

Ethnicity – CRIS

In SLaM source data, accessed via CRIS, patient ethnicity is recorded in structured fields and intended to be self-ascribed, although in practice, ethnicity may sometimes be assumed and ascribed by the clinician or other staff member completing the record. A patient's ethnicity may be recorded during their interactions with any SLaM service (including, but not limited to, CAMHS). CRIS aggregates ethnicity recorded in structured fields into 16+1 categories (Table S3), and this is the format that the ethnicity variable is

For peer review only - http://bmjopen.bmj.com/site/about/guidelines.xhtml

For peer review only

made available to researchers. For the pupils in this study, ethnicity was extracted from electronic health records via CRIS in March 2020.

To assist comparison with the NPD, we further aggregated CRIS ethnicity into White, Black, Asian, Chinese, Mixed, Other and Unknown (Table S4), although it should be noted that some inconsistencies between the resulting aggregate ethnic groups remain. Most notably, the NPD places Kurdish groups into the Other major ethnic group, while CRIS places them in the 16+1 category "any other White background", such that they were subsequently aggregated as White in CRIS. Also, the NPD places Malaysian groups into the Chinese major ethnic group, while CRIS places them in the 16+1 category "any other ethnic group", such that they were subsequently aggregated as Other in CRIS. Due to the aforementioned concerns around data security and confidentiality, we were unable to access more granular ethnicity variables to make these categorisations consistent between the data sources.

Statistical analysis

First, we summarised the completeness of ethnicity variables in the NPD and CRIS. We initially did this including the Unknown categories, then excluding these categories to determine the availability of what a previous study termed 'usable ethnicity' (16). Second, we used descriptive statistics and Cohen's kappa to explore how usable ethnicity compared between the NPD and CRIS for those who had ethnicity variables available from both data sources (23, 24).

We produced risk ratios to estimate how ethnicity from each data source was associated with the outcome variables of neurodevelopmental disorder and Year 11 educational attainment. Neurodevelopmental disorder was defined as any record of an intellectual disability (International Statistical Classification of Diseases and Related Health Problems – 10th Revision [ICD-10] codes F70x-F79x), pervasive developmental disorder

For peer review only - http://bmjopen.bmj.com/site/about/guidelines.xhtml

(ICD-10 codes F84x), or hyperkinetic disorder (ICD-10 codes F90x), derived from CRIS structured primary and secondary diagnosis fields (25). We focused on neurodevelopmental disorders because they are often pervasive and lifelong (26), therefore overcoming biases which might arise in differential timing of diagnoses. Year 11 educational attainment was derived from the NPD, and defined as achieving expected attainment thresholds of five A* to C General Certificates of Secondary Education (GCSEs) or equivalent grades including English and maths. These assessments typically take place at ages 15 to 16 years. A reduced sample of n=14,567 had available Year 11 attainment data and were included in these analyses, consistent with the fact that our cohort of pupils were age 4 to 18 at the time of linking their educational records, such that many had not yet completed their Year 11 assessments by the time of the linkage window. In order to understand how missing ethnicity data in either source might introduce bias into analyses of these variables, we combined all Unknown labels and empty cells into a single 'Missing' category.

Finally, in series of sensitivity analyses aiming to understand the impact of supplementing missing ethnicity data in one source using ethnicity data from the other source, we repeated these risk ratios, first using NPD ethnicity supplemented from CRIS when missing from the NPD, then using CRIS ethnicity supplemented from the NPD when missing from CRIS. All analyses were conducted in Stata 15.1 (27).

Results

Completeness of ethnicity variables

Including the Unknown categories, NPD ethnicity was available for n=25,951 (85.3%), and CRIS ethnicity was available for n=28,363 (93.2%). Excluding the Unknown categories, usable NPD ethnicity was available for n=24,127 (79.3%), and usable CRIS ethnicity was available for n=26,473 (87.0%) (Table 1). In total, n=29,609 (97.3%) had

For peer review only - http://bmjopen.bmj.com/site/about/guidelines.xhtml

usable ethnicity data from either the NPD or from CRIS, and n=20,991 (69.0%) had usable ethnicity data from both sources. Other sociodemographic information for the linked cohort has been reported elsewhere (20).

Table 1: Sample overview.

	n (%)
**Usable NPD-derived ethnicity, n=24,127**	
White	13,259 (55.0%)
Black	6,278 (26.0%)
Asian	755 (3.1%)
Chinese	73 (0.3%)
Mixed	3,247 (13.5%)
Other	515 (2.1%)
**Usable CRIS-derived ethnicity, n=26,473**	
White	14,572 (55.0%)
Black	7,400 (28.0%)
Asian	959 (3.6%)
Chinese	82 (0.3%)
Mixed	2,294 (8.7%)
Other	1,166 (4.4%)
**Year 11 expected attainment threshold achieved, n=14,567**	
No	10,009 (68.7%)
Yes	4,558 (31.3%)
**Neurodevelopmental disorder diagnosis, n=30,426**	
No	21,856 (71.8%)
Yes	8,570 (28.2%)

Comparability of aggregate ethnicity

Among pupils who had usable ethnicity data from both the NPD and CRIS (n=20,991), the two data sources agreed on 87.0% of categorisations, Cohen's kappa=0.79 (95% CI=0.78 to 0.79), indicating strong overall agreement.

Cross-tabulations between ethnicity data in the two data sources can be found in Table 2 (note that for the purposes of this table, we have excluded pupils who were

For peer review only - http://bmjopen.bmj.com/site/about/guidelines.xhtml

categorised as Chinese in either data source, in order to avoid disclosive cell counts). The majority of those categorised as White, Black, or Asian in the NPD were also categorised as such in CRIS (row percentages, Table 2), and vice versa (column percentages, Table 2). However, only 50.9% of those in the NPD's Mixed ethnic group were also classified as such in CRIS. Additionally, of those grouped as Other in CRIS, only 26.7% were also grouped as Other in the NPD; the same proportion were instead grouped as White (26.7%), and still more were grouped as Mixed (27.0%). Further detail on the proportion of disaggregated CRIS ethnicities categorised as consistent major ethnic groups in the NPD is available in Table S5.

For peer review only

For peer review only - http://bmjopen.bmj.com/site/about/guidelines.xhtml

Table 2: Cross-tabulations of CRIS and NPD ethnicity where ethnicity was available from both sources.

NPD		CRIS White n=11,850	CRIS Black n=5,743	CRIS Asian n=675	CRIS Mixed n=1,840	CRIS Other n=808	CRIS Total n=20,916
**White n=11,507**	Row %	96.1	0.7	0.2	1.2	1.9	100.0
	Column %	93.3	1.3	3.3	7.6	26.7	55.0
**Black n=5,532**	Row %	2.7	90.4	0.6	4.3	1.9	100.0
	Column %	1.3	87.1	5.2	13.0	13.2	26.5
**Asian n=631**	Row %	6.8	2.5	80.8	1.7	8.1	100.0
	Column %	0.4	0.3	75.6	0.6	6.3	3.0
**Mixed n=2,806**	Row %	17.3	21.0	3.1	50.9	7.8	100.0
	Column %	4.1	10.3	12.7	77.6	27.0	13.4
**Other n=440**	Row %	27.3	13.4	5.0	5.2	49.1	100.0
	Column %	1.0	1.0	3.3	1.3	26.7	2.1
**Total n=20,916**	Row %	56.7	27.5	3.2	8.8	3.9	100.0
	Column %	100.0	100.0	100.0	100.0	100.0	100.0

Note: For the purposes of this table, we have excluded pupils who were categorised as Chinese in either data source, in order to avoid disclosive cell counts.
Abbreviations: CRIS=Clinical Record Interactive Search, NPD=National Pupil Database.

For peer review only

Association with educational and clinical outcomes

The association between ethnicity, Year 11 attainment and neurodevelopmental disorder varied according to which data source was used to code ethnicity. Asian and Other ethnic groups were significantly more likely to achieve expected attainment thresholds in Year 11 than White ethnic groups when ethnicity was derived from the NPD, but not when ethnicity was derived from CRIS (Table 3). Also, compared to White ethnic groups, Mixed ethnic groups were less likely to receive neurodevelopmental disorder diagnoses only when

For peer review only - http://bmjopen.bmj.com/site/about/guidelines.xhtml

derived from the NPD, and Chinese ethnic groups were less likely to receive neurodevelopmental disorder diagnoses only when derived from CRIS (although it should be noted that the cell sizes underlying the risk ratios for the Chinese ethnic group were small) (Table 4). Associations with missing ethnicity data also varied according to which data source was used.

Finally, our sensitivity analyses indicated that supplementing missing ethnicity data from one source with ethnicity data from the other source brought these risk ratios more in line with each other to some degree. However, the strength of these risk ratios still varied according to whether CRIS or the NPD was used as the primary data source (Table S6, Table S7), and discrepancies in the estimated proportion of the sample belonging to each ethnic group also remained (Table S8).

For peer review only

For peer review only - http://bmjopen.bmj.com/site/about/guidelines.xhtml

Table 3: Unadjusted risk ratios between ethnicity derived from either NPD or CRIS (exposure) and whether the Year 11 expected attainment threshold was achieved (outcome; 'no' is the reference group)

	**NPD-derived ethnicity as exposure**				**CRIS-derived ethnicity as exposure**			
	**No (%)**	**Yes (%)**	**RR**	**95% CI**	**No (%)**	**Yes (%)**	**RR**	**95% CI**
**White**	4,485 (67.1%)	2,202 (32.9%)	Reference	-	5,041 (67.2%)	2,461 (32.8%)	Reference	-
**Black**	1,691 (69.6%)	739 (30.4%)	0.92	0.86 to 0.99	2,293 (75.1%)	761 (24.9%)	0.76	0.71 to 0.82
**Asian**	174 (52.4%)	158 (47.6%)	1.46	1.29 to 1.64	290 (63.5%)	167 (36.5%)	1.11	0.98 to 1.26
**Chinese**	15 (48.4%)	16 (51.6%)	1.59	1.05 to 2.13	17 (41.5%)	24 (58.5%)	1.78	1.31 to 2.20
**Mixed**	861 (66.3%)	438 (33.7%)	1.02	0.94 to 1.11	618 (69.1%)	276 (30.9%)	0.94	0.85 to 1.04
**Other**	110 (51.6%)	103 (48.4%)	1.48	1.27 to 1.70	369 (65.2%)	197 (34.8%)	1.06	0.94 to 1.19
**Missing**	2,673 (74.8%)	902 (25.2%)	0.76	0.71 to 0.82	1,381 (67.3%)	672 (32.7%)	1.00	0.93 to 1.07

Note: n=14,567. The n=15,859 not included in these analyses were missing data on Year 11 attainment.
Abbreviations: CI=Confidence Interval, CRIS=Clinical Record Interactive Search, NPD=National Pupil Database, RR=Risk Ratio.

For peer review only

Table 4: Unadjusted risk ratios between ethnicity derived from either NPD or CRIS (exposure) and neurodevelopmental disorder diagnosis (outcome; 'no' is the reference group)

	**NPD-derived ethnicity as exposure**				**CRIS-derived ethnicity as exposure**			
	**No (%)**	**Yes (%)**	**RR**	**95% CI**	**No (%)**	**Yes (%)**	**RR**	**95% CI**
**White**	9,218 (69.5%)	4,041 (30.5%)	Reference	-	9,963 (68.4%)	4,609 (31.6%)	Reference	-
**Black**	4,660 (74.2%)	1,618 (25.8%)	0.84	0.80 to 0.89	5,396 (72.9%)	2,004 (27.1%)	0.85	0.81 to 0.89
**Asian**	562 (74.4%)	193 (25.6%)	0.84	0.73 to 0.95	716 (74.7%)	243 (25.3%)	0.79	0.71 to 0.89
**Chinese**	53 (72.6%)	20 (27.4%)	0.90	0.60 to 1.28	65 (79.3%)	17 (20.7%)	0.65	0.41 to 0.98
**Mixed**	2,348 (72.3%)	899 (27.7%)	0.91	0.85 to 0.96	1,577 (68.7%)	717 (31.3%)	0.99	0.92 to 1.06
**Other**	425 (82.5%)	90 (17.5%)	0.57	0.47 to 0.69	908 (77.9%)	258 (22.1%)	0.69	0.61 to 0.77
**Missing**	4,590 (72.9%)	1,709 (27.1%)	0.89	0.84 to 0.93	3,231 (81.7%)	722 (18.3%)	0.57	0.53 to 0.61

Note: n=30,426.
Abbreviations: CI=Confidence Interval, CRIS=Clinical Record Interactive Search, NPD=National Pupil Database, RR=Risk Ratio.

For peer review only - http://bmjopen.bmj.com/site/about/guidelines.xhtml

Discussion

These findings suggest that linkage between health and education data sources can provide an opportunity to increase available ethnicity data for studies using linked and routinely collected records. In this sample, the proportion with available and usable ethnicity data rose from 79.3% (from the NPD) and 87.0% (from CRIS) to 97.3% (from either the NPD or from CRIS). Ethnicity at an aggregate level mostly aligned between the two data sources, with the NPD and CRIS agreeing on 87.0% of classifications. However, as with previous studies, some divergence was observed, particularly in the Mixed and Other ethnic groups (16).

Low quality ethnicity data is known to pose a challenge for investigating ethnic inequalities in public health (5). A previous United Kingdom study found incomplete and inconsistent ethnicity recordings in two health datasets (16), and outputs accessed via the United Kingdom government's online data service corroborate that collection and reporting of ethnicity data within the public sector is not consistent (28).

This study updates and extends previous findings by comparing two health and education datasets, thereby taking a cross-disciplinary perspective on this issue. It also demonstrates that many of the same measurement issues effecting ethnicity data in other datasets and populations can be present in cross-disciplinary datasets for children and adolescents accessing mental health services. This is important because many research studies and national statistical releases make use of routinely-collected ethnicity data to estimate inequalities affecting CAMHS (e.g. inequalities in referral pathways) and affecting the education system (e.g. inequalities in school exclusions) (29, 30).

Consistent with previous work in other populations, we found that linkage between these datasets increased availability of ethnicity data, but that the datasets did not consistently agree on ethnicity, particularly for Mixed and Other ethnic groups. The Other ethnic category

For peer review only - http://bmjopen.bmj.com/site/about/guidelines.xhtml

is known to capture particularly heterogenous groups, such that non-alignment on this category is perhaps unsurprising. Indeed, the Other classification in each source may partly comprise individuals who do not feel that their ethnicity is otherwise captured by options offered by the data collection procedure. For example, when submitting ethnicity data for the school census, a range of ethnicity options are offered by the NPD which would ultimately be aggregated as White (such as Cornish, Italian, White Eastern European and White Western European). However, these options are not available in CRIS, such that in the clinical setting the patient, parent or recording clinician might instead select "any other group" as an alternative and be aggregated as Other. Disagreements between the data sources on the Mixed ethnic group might reflect previous United Kingdom research findings that individuals from Mixed ethnic groups are culturally positioned between White and minority ethnic groups, and how they self-identify may be particularly fluid and context-dependent (31, 32).

Therefore, some inconsistencies in ethnicity between multiple data sources are expected. Ethnicity is not a time-invariant characteristic, is highly context specific, and self-reported ethnic identity has been known to vary in the same individuals over the life course (33). However, our findings suggest that this can result in substantial differences in estimated associations between ethnicity and important clinical and educational outcomes.

Our findings pose serious challenges for accurately identifying inequalities which need to be addressed. A recent report recommended the enforcement of guidelines to ensure that patient ethnicity is recorded, and recorded accurately based on self-report, during interactions with healthcare services. Doing so might resolve some of the observed discrepancies (5), particularly as our study indicated large differences in observed associations for children with 'missing' ethnicity. However, it is important to note that both the coverage and quality of ethnicity recording in routinely collected data need to improve; solely enforcing more comprehensive completion of ethnicity fields could cause the quality

For peer review only - http://bmjopen.bmj.com/site/about/guidelines.xhtml

and accuracy of these data to deteriorate further if not accompanied by updated guidance on recording ethnicity (34).

Some of the issues we identified might also be resolved by data sources supplying ethnicity data to researchers at a more granular level to ensure consistency in how ethnicity is aggregated. Understandably, concerns around data anonymity sometimes preclude this, but in light of known inequalities at the level of more granular ethnicity and growing ‘super-diversity’ in the United Kingdom, aggregate ethnicity data remain insufficient (35, 36). Where aggregation is essential for maintaining anonymity, other methodological improvements might include standardisation across the public sector in how ethnicity is aggregated, or allowing researchers to specify how they want ethnicity to be aggregated when requesting data extracts. Researchers reporting analyses from linked datasets should also be transparent about how they have coded ethnicity, and might consider conducting sensitivity analyses to understand the possible impact of these coding decisions on their analyses.

Efforts to effectively capture ethnicity in research are ongoing. The Office for National Statistics conducted a public consultation and stakeholder survey to inform ethnic group data collection techniques in preparation for the 2021 Census. Their findings indicated that aggregated ethnicity categories (such as White, Black, Asian, Mixed and Other) do not sufficiently meet the requirements of many data users (37). Our findings reinforce that not only is aggregate ethnicity data insufficient, but that it does not always align between large-scale data sources. Moreover, choosing which data source to primarily code ethnicity from could have substantial implications for how associations between ethnicity, health and educational outcomes are interpreted, such that users of linked data should be cautious in combining and comparing ethnicity between different data sources.

We used broad terminology in reference to ethnicity, which is not desirable; however, as discussed, a more specific examination and discussion was precluded by only having

For peer review only - http://bmjopen.bmj.com/site/about/guidelines.xhtml

access to aggregate ethnicity variables (6). Additionally, the terminology follows Office for National Statistics standard approaches for aggregation which are commonly used by researchers and government analysts to inform United Kingdom policy. This is helpful for generalisability.

We only used one assessment of ethnicity per individual and per data source, and we were unable to ascertain exactly when ethnicity was ascribed. Therefore within-person changes in reported ethnicity over time could not be assessed.

We did not adjust the regression models for key covariates such as socioeconomic status, gender and age. It should be emphasised that our aim was not to provide a precise estimate of the association between ethnicity and attainment or neurodevelopmental disorder, but to ascertain whether variable findings can arise when measuring ethnicity from different data sources. Moreover, gender, socioeconomic status, and age were also recorded inconsistently between the datasets, so including them would raise further questions as to which data source to rely on for their classification: this is beyond the scope of this study's focus on discrepancies seen in ethnicity. However, whether these characteristics in turn predict ethnicity misclassification would be an area for future research. We also did not consider other important characteristics such as religion, first language and country of birth – future work could consider these characteristics to investigate whether linkage might inform inequalities research from an intersectional perspective (38).

Conclusion

Data linkage minimises missingness in ethnicity data, and ethnicity categorisations between CRIS and the NPD were mostly consistent. However, choosing which data source to primarily code ethnicity from can impact findings relating to ethnicity, mental health, and education.

For peer review only - http://bmjopen.bmj.com/site/about/guidelines.xhtml

Funding statement

This paper represents independent research funded by the National Institute for Health Research (NIHR) Biomedical Research Centre at South London and Maudsley NHS Foundation Trust and King's College London (NIHR-INF-0690). The views expressed are those of the authors and not necessarily those of the NIHR, the Department of Health and Social Care or King’s College London.

Competing interests

AW is supported by ADR UK (Administrative Data Research UK), an Economic and Social Research Council (ESRC) investment (part of UK Research and Innovation) (ES/W002531/1). RS is part-funded by: i) the National Institute for Health Research (NIHR) Biomedical Research Centre at the South London and Maudsley NHS Foundation Trust and King’s College London; ii) the National Institute for Health Research (NIHR) Applied Research Collaboration South London (NIHR ARC South London) at King’s College Hospital NHS Foundation Trust; iii) the DATAMIND HDR UK Mental Health Data Hub (MRC grant MR/W014386). RS has received research support in the last 3 years from Janssen, GSK and Takeda. JD is supported by NIHR Clinician Science Fellowship award (CS-2018-18-ST2-014) and has received support from a Medical Research Council (MRC) Clinical Research Training Fellowship (MR/L017105/1) and Psychiatry Research Trust Peggy Pollak Research Fellowship in Developmental Psychiatry. JDM is supported by the ESRC Centre for Society and Mental Health at King’s College London (ESRC Reference: ES/S012567/1), grants from the ESRC (ES/S002715/1) and by the National Institute for Health Research (NIHR) Biomedical Research Centre at South London and Maudsley NHS Foundation Trust and King's College London and the National Institute for Health Research (NIHR) Applied Research Collaboration South London (NIHR ARC South London) at King’s College Hospital NHS Foundation Trust. The views expressed are those of the authors

For peer review only - http://bmjopen.bmj.com/site/about/guidelines.xhtml

and not necessarily those of the ESRC, NIHR, the Department of Health and Social Care or King's College London.

Author contributions

AW conceived and designed the study, performed data analysis, and wrote the manuscript. JD led the data linkage, conducted initial cleaning of NPD data, and provided critical comments on the manuscript. AJ performed data extraction. JDM, TF, and RS consulted on study design and interpretation, and provided critical comments on the manuscript. All authors read and approved the final manuscript. At the time of undertaking this study, JD and AW had full access to the dataset from which the study population was derived.

Data sharing statement

The data that support the findings of this study are available from the Department for Education and South London and Maudsley NHS Foundation Trust, but restrictions apply to the availability of these data, and so they are not publicly available. For the purposes of open access, the author has applied a Creative Commons Attribution (CC BY) licence to any Accepted Author Manuscript version arising from this submission.

Ethical approval statement

The CRIS data resource, including the linked data used in this manuscript, has received research ethics approval for secondary analyses (Oxford REC C, reference 18/SC/0372). The CRIS Oversight Committee ensures that research conducted using the health records are ethical and legal, and service users can opt-out of their CRIS data being used for research.

For peer review only - http://bmjopen.bmj.com/site/about/guidelines.xhtml

References

1. Department for Education. Key stage 4 performance 2019 (revised). London, United Kingdom; 2020.
2. Wickersham A, Dickson H, Jones R, Pritchard M, Stewart R, Ford T, et al. Educational attainment trajectories among children and adolescents with depression, and the role of sociodemographic characteristics: longitudinal data-linkage study. *The British Journal of Psychiatry*. 2020;218(3):151-157. doi:10.1192/bjp.2020.160
3. Wickersham A, Ford T, Stewart R, Downs J. Estimating the impact of child and early adolescent depression on subsequent educational attainment: secondary analysis of an existing data linkage. *Epidemiology and Psychiatric Sciences*. 2021;30:e76. doi:10.1017/S2045796021000603
4. Tromans S, Chester V, Gemegah E, Roberts K, Morgan Z, Yao GL, et al. Autism identification across ethnic groups: a narrative review. *Advances in Autism*. 2020;7(3):241-255. doi:10.1108/AIA-03-2020-0017
5. Kapadia D, Zhang J, Salway S, Nazroo J, Booth A, Villarroel-Williams N, et al. Ethnic Inequalities in Healthcare: A Rapid Evidence Review. London, United Kingdom; 2022.
6. Bhopal R. Glossary of terms relating to ethnicity and race: for reflection and debate. *Journal of Epidemiology & Community Health*. 2004;58(6):441-445. doi:10.1136/jech.2003.013466
7. Hickman MJ, Ryan L. The "Irish question": marginalizations at the nexus of sociology of migration and ethnic and racial studies in Britain. *Ethnic and Racial Studies*. 2020;43(16):96-114. doi:10.1080/01419870.2020.1722194
8. Senior PA, Bhopal R. Ethnicity as a variable in epidemiological research. *BMJ*. 1994;309:327. doi:10.1136/bmj.309.6950.327
9. Boehmer U, Kressin NR, Berlowitz DR, Christiansen CL, Kazis LE, Jones JA. Self-reported vs administrative race/ethnicity data and study results. *American Journal of Public Health*. 2002;92(9):1471-1472. doi:10.2105/AJPH.92.9.1471
10. Gialamas A, Pilkington R, Berry J, Scalzi D, Gibson O, Brown A, et al. Identification of Aboriginal children using linked administrative data: consequences for measuring inequalities. *Journal of Paediatrics and Child Health*. 2016;52(5):534-540.
11. Lawrence D, Christensen D, Mitrou F, Draper G, Davis G, McKeown S, et al. Adjusting for under-identification of Aboriginal and/or Torres Strait Islander births in time series produced from birth records: Using record linkage of survey data and administrative data sources. *BMC Medical Research Methodology*. 2012;12(1):90. doi:10.1186/1471-2288-12-90
12. López MM, Bevans M, Wehrlen L, Yang L, Wallen G. Discrepancies in race and ethnicity documentation: a potential barrier in identifying racial and ethnic disparities. *Journal of Racial and Ethnic Health Disparities*. 2017;4:812-818. doi:10.1007/s40615-016-0283-3
13. McAlpine DD, Beebe TJ, Davern M, Call KT. Agreement between self-reported and administrative race and ethnicity data among Medicaid enrollees in Minnesota. *Health services research*. 2007;42(6p2):2373-2388. doi:10.1111/j.1475-6773.2007.00771.x
14. Saunders CL, Abel GA, El Turabi A, Ahmed F, Lyratzopoulos G. Accuracy of routinely recorded ethnic group information compared with self-reported ethnicity: evidence from the English Cancer Patient Experience survey. *BMJ Open*. 2013;3:e002882. doi:10.1136/bmjopen-2013-002882
15. Scobie S, Spencer J, Raleigh V. Ethnicity coding in English health service datasets. London, United Kingdom2021. [Available from: https://www.nuffieldtrust.org.uk/research/ethnicity-coding-in-english-health-service-datasets, accessed 11 November 2021].
16. Mathur R, Bhaskaran K, Chaturvedi N, Leon DA, vanStaa T, Grundy E, et al. Completeness and usability of ethnicity data in UK-based primary care and hospital databases. *Journal of Public Health*. 2013;36(4):684-692. doi:10.1093/pubmed/fdt116
17. Wickersham A, Downs J. Clinical applications of big data to child and adolescent mental health care. Shaping the Future of Child and Adolescent Mental Health: Elsevier; 2023. p. 59-79.

For peer review only - http://bmjopen.bmj.com/site/about/guidelines.xhtml

18. Office for National Statistics. 2011 census: ethnic group (detailed), local authorities in England and Wales. 2011 Census: Key Statistics and Quick Statistics for Wards and Output Areas in England and Wales2013.
19. Downs J, Gilbert R, Hayes RD, Hotopf M, Ford T. Linking health and education data to plan and evaluate services for children. *Archives of Disease in Childhood*. 2017;102(7):599-602. doi:10.1136/archdischild-2016-311656
20. Downs J, Ford T, Stewart R, Epstein S, Shetty H, Little R, et al. An approach to linking education, social care and electronic health records for children and young people in South London: a linkage study of child and adolescent mental health service data. *BMJ Open*. 2019;9(1):e024355. doi:10.1136/bmjopen-2018-024355
21. Jay MA, Mc Grath-Lone L, Gilbert R. Data resource: the National Pupil Database (NPD). *International Journal of Population Data Science*. 2019;4(1). doi:10.23889/ijpds.v4i1.1101
22. Office for National Statistics. Ethnic group, national identity and religion. 2016. [Available from: https://www.ons.gov.uk/methodology/classificationsandstandards/measuringequality/ethnicgroupnationalidentityandreligion, accessed 07 September 2020].
23. Cohen J. A coefficient of agreement for nominal scales. *Educational and Psychological Measurement*. 1960;20(1):37-46. doi:10.1177/001316446002000104
24. McHugh ML. Interrater reliability: the kappa statistic. *Biochemia Medica*. 2012;22(3):276-282. doi:10.11613/BM.2012.031
25. World Health Organization. International statistical classification of diseases and related health problems - 10th revision, edition 2010. World Health Organization; 2011. [Available from: https://icd.who.int/browse10/2010/en, accessed 12 October 2021].
26. Thapar A, Cooper M, Rutter M. Neurodevelopmental disorders. *The Lancet Psychiatry*. 2017;4(4):339-346. doi:10.1016/S2215-0366(16)30376-5
27. StataCorp. Stata Statistical Software: Release 15. College Station, TX: StataCorp LLC; 2017.
28. Cabinet Office. The 2021 census of population and housing in England and Wales. 2014. [Available from: https://www.gov.uk/government/publications/the-2021-census-of-population-and-housing-in-england-and-wales, accessed
29. Chui Z, Gazard B, MacCrimmon S, Harwood H, Downs J, Bakolis I, et al. Inequalities in referral pathways for young people accessing secondary mental health services in south east London. *European Child & Adolescent Psychiatry*. 2020;30:1113–1128. doi:10.1007/s00787-020-01603-7
30. Office for National Statistics. Permanent exclusions and suspensions in England. 2023. [Available from: https://explore-education-statistics.service.gov.uk/find-statistics/permanent-and-fixed-period-exclusions-in-england/2021-22-summer-term, accessed 07 November 2023].
31. Barrett M, Eade J, Cinnirella M, Garbin D. New ethnicities among British Bangladeshi and mixed-heritage youth. 2006.
32. Panico L, Nazroo JY. The social and economic circumstances of mixed ethnicity children in the UK: findings from the Millennium Cohort Study. *Ethnic and Racial Studies*. 2011;34(9):1421-1444. doi:10.1080/01419870.2011.556745
33. Simpson L. How have people's ethnic identities changed in England and Wales? Manchester, United Kingdom; 2014.
34. Raleigh V, Goldblatt P. Ethnicity coding in health records. Correspondence to NHS England2020. [Available from: https://www.kingsfund.org.uk/publications/ethnicity-coding-health-records, accessed 01 April 2022].
35. Strand S. Ethnicity, gender, social class and achievement gaps at age 16: intersectionality and 'getting it' for the white working class. *Research Papers in Education*. 2014;29(2):131-171. doi:10.1080/02671522.2013.767370
36. Vertovec S. Super-diversity and its implications. *Ethnic and Racial Studies*. 2007;30(6):1024-1054. doi:10.1080/01419870701599465

For peer review only - http://bmjopen.bmj.com/site/about/guidelines.xhtml

37. Office for National Statistics. 2021 census: ethnic group stakeholder follow-up survey – summary of responses. 2019. [Available from: https://www.ons.gov.uk/census/censustransformationprogramme/questiondevelopment/ethnicgroupnationalidentityreligionandlanguage/2021censusethnicgroupstakeholderfollowupsurveysummaryofresponses, accessed 07 September 2020].

38. Gazard B, Frissa S, Nellums L, Hotopf M, Hatch SL. Challenges in researching migration status, health and health service use: an intersectional analysis of a South London community. *Ethnicity & Health*. 2015;20(6):564-593. doi:10.1080/13557858.2014.961410

For peer review only

Table S1: The RECORD statement – checklist of items, extended from the STROBE statement, that should be reported in observational studies using routinely collected health data. Items specific to cross-sectional/case-control studies have been removed.

	Item No.	STROBE items	Page	RECORD items	Page
**Title and abstract**					
	1	(a) Indicate the study's design with a commonly used term in the title or the abstract (b) Provide in the abstract an informative and balanced summary of what was done and what was found	2	RECORD 1.1: The type of data used should be specified in the title or abstract. When possible, the name of the databases used should be included.<br><br>RECORD 1.2: If applicable, the geographic region and timeframe within which the study took place should be reported in the title or abstract.<br><br>RECORD 1.3: If linkage between databases was conducted for the study, this should be clearly stated in the title or abstract.	2
**Introduction**					
Background rationale	2	Explain the scientific background and rationale for the investigation being reported	3-5	N/A	
Objectives	3	State specific objectives, including any prespecified hypotheses	3-5	N/A	
**Methods**					
Study Design	4	Present key elements of study design early in the paper	5	N/A	
Setting	5	Describe the setting, locations, and relevant dates, including periods of recruitment, exposure, follow-up, and data collection	5	N/A	
Participants	6	(a) Cohort study - Give the eligibility criteria, and the sources and methods of selection of participants. Describe methods of follow-up<br><br>(b) Cohort study - For matched studies, give matching criteria and number of exposed and unexposed	5	RECORD 6.1: The methods of study population selection (such as codes or algorithms used to identify subjects) should be listed in detail. If this is not possible, an explanation should be provided.<br><br>RECORD 6.2: Any validation studies of the codes or algorithms used to select the population should be referenced. If validation	5

For peer review only

	Item No.	STROBE items	Page	RECORD items	Page
				was conducted for this study and not published elsewhere, detailed methods and results should be provided.<br><br>RECORD 6.3: If the study involved linkage of databases, consider use of a flow diagram or other graphical display to demonstrate the data linkage process, including the number of individuals with linked data at each stage.	
Variables	7	Clearly define all outcomes, exposures, predictors, potential confounders, and effect modifiers. Give diagnostic criteria, if applicable.	6-8	RECORD 7.1: A complete list of codes and algorithms used to classify exposures, outcomes, confounders, and effect modifiers should be provided. If these cannot be reported, an explanation should be provided.	6-8
Data sources/ measurement	8	For each variable of interest, give sources of data and details of methods of assessment (measurement).<br>Describe comparability of assessment methods if there is more than one group	6-8	N/A	
Bias	9	Describe any efforts to address potential sources of bias	6-8	N/A	
Study size	10	Explain how the study size was arrived at	5	N/A	
Quantitative variables	11	Explain how quantitative variables were handled in the analyses. If applicable, describe which groupings were chosen, and why	N/A	N/A	
Statistical methods	12	(a) Describe all statistical methods, including those used to control for confounding<br>(b) Describe any methods used to examine subgroups and interactions<br>(c) Explain how missing data were addressed<br>(d) Cohort study - If applicable, explain how loss to follow-up was addressed<br>(e) Describe any sensitivity analyses	7-8	N/A	
Data access and cleaning methods		N/A		RECORD 12.1: Authors should describe the extent to which the investigators had access to the database population used to create the study population.	5, 20

For peer review only

	Item No.	STROBE items	Page	RECORD items	Page
				RECORD 12.2: Authors should provide information on the data cleaning methods used in the study.	
Linkage		N/A		RECORD 12.3: State whether the study included person-level, institutional-level, or other data linkage across two or more databases. The methods of linkage and methods of linkage quality evaluation should be provided.	5
**Results**					
Participants	13	(a) Report the numbers of individuals at each stage of the study (e.g., numbers potentially eligible, examined for eligibility, confirmed eligible, included in the study, completing follow-up, and analysed)<br>(b) Give reasons for non-participation at each stage.<br>(c) Consider use of a flow diagram	Supplement	RECORD 13.1: Describe in detail the selection of the persons included in the study (i.e., study population selection) including filtering based on data quality, data availability and linkage. The selection of included persons can be described in the text and/or by means of the study flow diagram.	5
Descriptive data	14	(a) Give characteristics of study participants (e.g., demographic, clinical, social) and information on exposures and potential confounders<br>(b) Indicate the number of participants with missing data for each variable of interest<br>(c) Cohort study - summarise follow-up time (e.g., average and total amount)	9	N/A	
Outcome data	15	Cohort study - Report numbers of outcome events or summary measures over time	9	N/A	
Main results	16	(a) Give unadjusted estimates and, if applicable, confounder-adjusted estimates and their precision (e.g., 95% confidence interval). Make clear which confounders were adjusted for and why they were included<br>(b) Report category boundaries when continuous variables were categorized<br>(c) If relevant, consider translating estimates of relative risk into absolute risk for a meaningful time period	11-14	N/A	

For peer review only

	Item No.	STROBE items	Page	RECORD items	Page
Other analyses	17	Report other analyses done—e.g., analyses of subgroups and interactions, and sensitivity analyses	12	N/A	
**Discussion**					
Key results	18	Summarise key results with reference to study objectives	15-17	N/A	
Limitations	19	Discuss limitations of the study, taking into account sources of potential bias or imprecision. Discuss both direction and magnitude of any potential bias	17-18	RECORD 19.1: Discuss the implications of using data that were not created or collected to answer the specific research question(s). Include discussion of misclassification bias, unmeasured confounding, missing data, and changing eligibility over time, as they pertain to the study being reported.	17-18
Interpretation	20	Give a cautious overall interpretation of results considering objectives, limitations, multiplicity of analyses, results from similar studies, and other relevant evidence	15-17	N/A	
Generalisability	21	Discuss the generalisability (external validity) of the study results	18	N/A	
**Other Information**					
Funding	22	Give the source of funding and the role of the funders for the present study and, if applicable, for the original study on which the present article is based	19	N/A	
Accessibility of protocol, raw data, and programming code		N/A		RECORD 22.1: Authors should provide information on how to access any supplemental information such as the study protocol, raw data, or programming code.	20

*Reference: Benchimol, E. I., Smeeth, L., Guttmann, A., Harron, K., Moher, D., Petersen, I., ... & RECORD Working Committee. (2015). The REporting of studies Conducted using Observational Routinely-collected health Data (RECORD) statement. PLoS Med, 12(10), e1001885.

*Checklist is protected under Creative Commons Attribution (CC BY) license.

Figure S1: Study flow diagram.

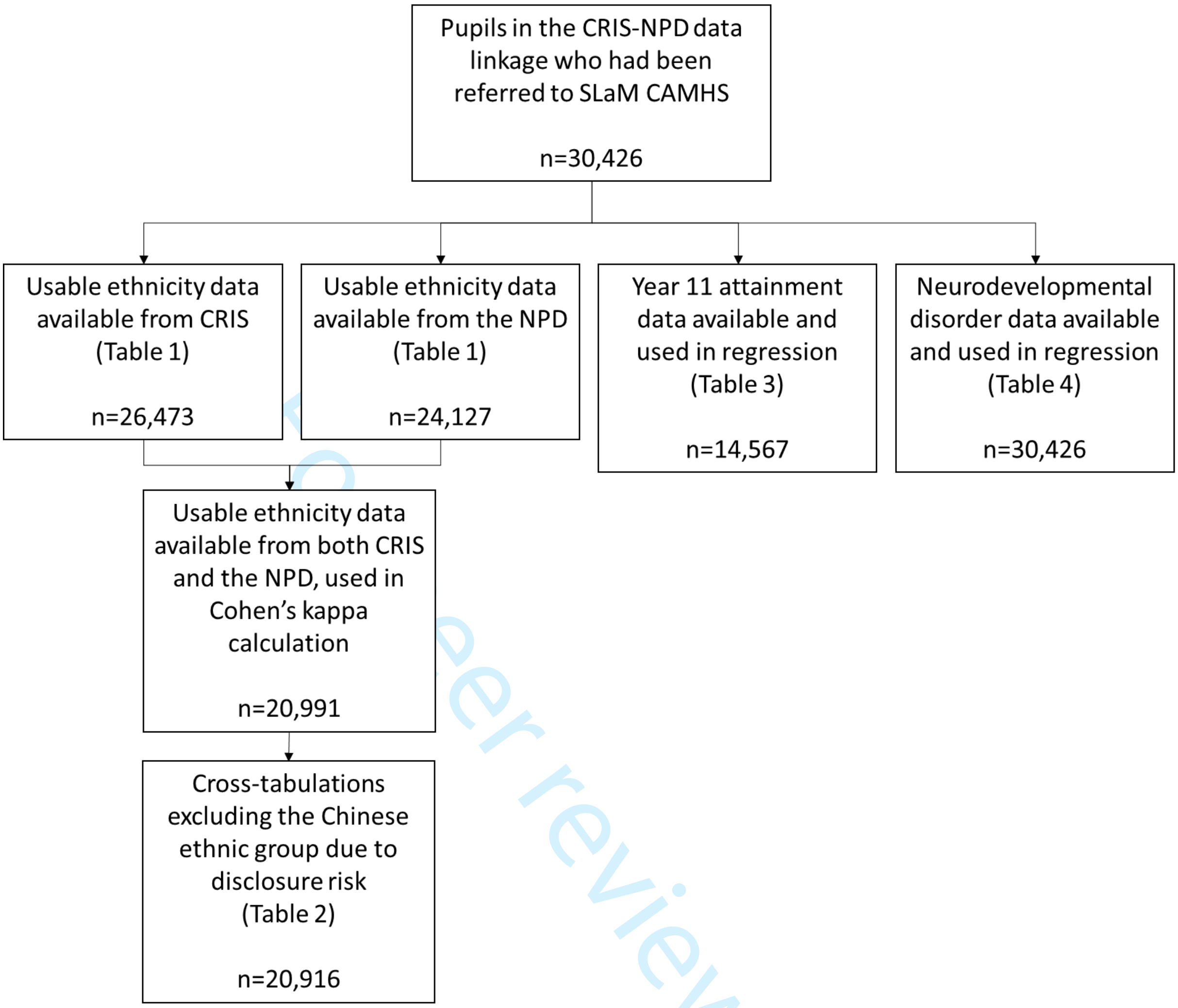

For peer review only

Table S2: Ethnic groups in the NPD.

Approved extended categories	Minor ethnic group	Major ethnic group
White – British	White - British	White
White – Cornish [a]		
White – English		
White – Scottish		
White – Welsh		
Other White British		
White – Irish	White	
Traveller of Irish heritage		
Any other White background	Any other White background	
Albanian		
Bosnian-Herzegovinian		
Croatian		
Greek/Greek Cypriot		
Greek		
Greek Cypriot		
Italian [a]		
Kosovan		
Portuguese		
Serbian		
Turkish/Turkish Cypriot		
Turkish		
Turkish Cypriot		
White European [a]		
White Eastern European [a]		
White Western European [a]		
White other		
Gypsy/Roma	Gypsy/Roma	
Gypsy		
Roma		
Other Gypsy/Roma		
White and Black Caribbean	White and Black Caribbean	Mixed/Dual background
White and Black African	White and Black African	
White and Asian	White and Asian	
White and Pakistani [a]		
White and Indian [a]		
White and any other Asian background		
Any other Mixed background	Any other Mixed background	
Asian and any other ethnic group [a]		
Asian and Black		
Asian and Chinese		
Black and any other ethnic group [a]		
Black and Chinese		

For peer review only

Approved extended categories	Minor ethnic group	Major ethnic group
Chinese and any other ethnic group [a]		
White and any other ethnic group [a]		
White and Chinese		
Other Mixed background		
Indian	Indian	Asian or Asian British
Pakistani	Pakistani	
Mirpuri Pakistani [a]		
Kashmiri Pakistani [a]		
Other Pakistani		
Bangladeshi	Bangladeshi	
Any other Asian background	Any other Asian background	
African Asian [a]		
Kashmiri other [a]		
Nepali [a]		
Sri Lankan Sinhalese		
Sri Lankan Tamil		
Sri Lankan other		
Other Asian		
Black Caribbean	Black Caribbean	Black or Black British
Black - African	Black - African	
Black - Angolan		
Black - Congolese [a]		
Black – Ghanaian		
Black – Nigerian		
Black - Sierra Leonean [a]		
Black – Somali		
Black – Sudanese		
Other Black African		
Any other Black background	Any other Black background	
Black European [a]		
Black North American [a]		
Other Black		
Chinese	Chinese	Chinese
Hong Kong Chinese [a]		
Malaysian Chinese		
Singaporean Chinese [a]		
Taiwanese [a]		
Other Chinese		
Any other ethnic group	Any other ethnic group	Any other ethnic group
Afghan [a]		
Arab other		
Egyptian [a]		
Filipino		
Iranian		

For peer review only

Approved extended categories	Minor ethnic group	Major ethnic group
Iraqi		
Japanese		
Korean [a]		
Kurdish		
Latin/South/Central American [a]		
Lebanese [a]		
Libyan [a]		
Malay		
Moroccan [a]		
Polynesian [a]		
Thai [a]		
Vietnamese		
Yemeni		
Other ethnic group		
Refused	Refused	Refused
Information not yet obtained	Information not yet obtained	Information not yet obtained

a. Specific ethnicities not included in the SLaM electronic health record ethnic categories list (CRIS, Supplement 4).

Note: Contains public sector information licensed under the Open Government Licence v3.0.

For peer review only

Table S3: Ethnic groups in CRIS.

SLaM electronic health record ethnic categories	16+1 ethnic categories supplied from CRIS
British (A)	British (A)
English (CA)	
Scottish (CB)	
Welsh (CC)	
Irish (B)	Irish (B)
Irish Traveller (CL)	
Albanian (CS)	Any other White background €
All former USSR Republics (CQ) [a]	
Bosnian (CT)	
Croatian (CU)	
Cypriot (part not stated) (CE) [a]	
Greek (CF)	
Greek Cypriot (CG)	
Gypsy/Romany (CN)	
Kosovan (CR)	
Kurdish (C5)	
Other former Yugoslavia (CW) [a]	
Other White Unspecified (C3)	
Other White/Mixed European (CY) [a]	
Portuguese (C4)	
Serbian (CV)	
Traveller (CM) [a]	
Turkish (CH)	
Turkish Cypriot (CJ)	
White and Black Caribbean (D)	White and Black Caribbean (D)
White and Black African €	White and Black African €
White and Asian (F)	White and Asian (F)
Asian and Chinese (GE)	Any other Mixed background (G)
Black and Asian (GA)	
Black and Chinese (GB)	
Black and White (GC) [a]	
Chinese and White (GD)	
Indian/British Indian (H)	Indian (H)
Pakistani/British Pakistani (J)	Pakistani (J)
Bangladeshi/British Bangladeshi (K)	Bangladeshi (K)
British Asian (LH) [a]	Any other Asian background (L)
Caribbean Asian (LJ) [a]	
East African Asian (LD) [a]	
Mixed Asian (LA) [a]	
Other Asian Unspecified (LK)	
Sinhalese (LG)	
Sri Lankan (LE)	
Tamil (LF)	

For peer review only

SLaM electronic health record ethnic categories	16+1 ethnic categories supplied from CRIS
Caribbean (M)	Caribbean (M)
Algerian (PP) [a]<br>Angolan (PJ)<br>Eritrean (PK) [a]<br>Ethiopian (PL) [a]<br>Ghanaian (PM)<br>Nigerian (PC)<br>Somali (PA)<br>Sudanese (PH)<br>Ugandan (PQ) [a]<br>Other African (N)	African (N)
Mixed Black (PB) [a]<br>Black British (PD) [a]<br>Other Black Unspecified (PE)	Any other Black background (P)
Chinese (R)	Chinese (R)
Any Other Group (SE)<br>Arab (SG)<br>Columbian (SK) [a]<br>Ecuadorian (SL) [a]<br>Filipino (SC)<br>Iranian (SH)<br>Iraqi (SJ)<br>Japanese (SB)<br>Malaysian (SD)<br>Middle Eastern (SF) [a]<br>Other Latin American (SM)<br>Vietnamese (SA)	Any other ethnic group (S)
Not Stated (Z)	Not Stated (Z)

a. Specific ethnicities not included in the ‘approved extended ethnic categories’ list (NPD, Supplement 3).

For peer review only

Table S4: Aggregating CRIS ethnicity to assist comparison with the NPD.

16+1 ethnic categories supplied from CRIS	Aggregate category
British (A)	White
Irish (B)	
Any other White background (C)	
White and Black Caribbean (D)	Mixed
White and Black African (E)	
White and Asian (F)	
Any other Mixed background (G)	
Indian (H)	Asian
Pakistani (J)	
Bangladeshi (K)	
Any other Asian background (L)	
Caribbean (M)	Black
African (N)	
Any other Black background (P)	
Chinese (R)	Chinese
Any other ethnic group (S)	Other
Not Stated (Z)	Unknown

For peer review only

Table S5: Proportion of disaggregated CRIS ethnicities categorised as consistent major ethnic groups in the NPD, among individuals where ethnicity was available from both sources (n=20,916).

		NPD				
		White	Black	Asian	Mixed	Other
CRIS	British	94.6%				
	Irish	93.8%				
	Any other White background	80.5%				
	African		91.6%			
	Caribbean		90.2%			
	Any other Black background		84.3%			
	Indian			83.5%		
	Pakistani			89.9%		
	Bangladeshi			94.6%		
	Any other Asian background			55.5%		
	White and Asian				77.6%	
	White and Black African				75.2%	
	White and Black Caribbean				79.2%	
	Any other Mixed background				74.3%	
	Any other ethnic group					26.7%

Note: Row percentages provided. Off-diagonal proportions have been suppressed to avoid disclosive cell counts. For the purposes of this table, we have excluded pupils who were categorised as Chinese in either data source, in order to avoid disclosive cell counts.

For peer review only

Table S6: Unadjusted risk ratios between ethnicity derived from either NPD or CRIS (exposure) and whether the Year 11 expected attainment threshold was achieved (outcome; 'no' is the reference group)

	NPD-derived ethnicity as exposure, supplemented from CRIS if missing				CRIS-derived ethnicity as exposure, supplemented from NPD if missing			
	**No (%)**	**Yes (%)**	**RR**	**95% CI**	**No (%)**	**Yes (%)**	**RR**	**95% CI**
**White**	5,641 (67.8%)	2,682 (32.2%)	Reference	-	5,725 (67.4%)	2,765 (32.6%)	Reference	-
**Black**	2,420 (73.2%)	885 (26.8%)	0.83	0.78 to 0.89	2,473 (73.5%)	891 (26.5%)	0.81	0.76 to 0.87
**Asian**	311 (61.1%)	198 (38.9%)	1.21	1.07 to 1.34	317 (62.4%)	191 (37.6%)	1.15	1.02 to 1.29
**Chinese**	25 (53.2%)	22 (46.8%)	1.45	1.03 to 1.88	18 (40.9%)	26 (59.1%)	1.81	1.35 to 2.22
**Mixed**	1,002 (67.7%)	478 (32.3%)	1.00	0.92 to 1.08	758 (69.1%)	339 (30.9%)	0.95	0.86 to 1.04
**Other**	280 (63.9%)	158 (36.1%)	1.12	0.98 to 1.26	388 (64.8%)	211 (35.2%)	1.08	0.96 to 1.21
**Missing**	330 (71.0%)	135 (29.0%)	0.90	0.78 to 1.04	330 (71.0%)	135 (29.0%)	0.89	0.77 to 1.03

Note: n=14,567. The n=15,859 not included in these analyses were missing data on Year 11 attainment. The n=817 in the missing ethnicity group are those with ethnicity data available neither in CRIS, nor the NPD.
Abbreviations: CI=Confidence Interval, CRIS=Clinical Record Interactive Search, NPD=National Pupil Database, RR=Risk Ratio.

For peer review only

Table S7: Unadjusted risk ratios between ethnicity derived from either NPD or CRIS (exposure) and neurodevelopmental disorder diagnosis (outcome; 'no' is the reference group)

	NPD-derived ethnicity as exposure, supplemented from CRIS if missing				CRIS-derived ethnicity as exposure, supplemented from NPD if missing			
	**No (%)**	**Yes (%)**	**RR**	**95% CI**	**No (%)**	**Yes (%)**	**RR**	**95% CI**
**White**	11,098 (69.5%)	4,880 (30.5%)	Reference	-	11,377 (69.7%)	4,947 (30.3%)	Reference	-
**Black**	5,896 (74.3%)	2,039 (25.7%)	0.84	0.80 to 0.88	6,017 (73.9%)	2,129 (26.1%)	0.86	0.82 to 0.90
**Asian**	782 (75.5%)	254 (24.5%)	0.80	0.71 to 0.89	816 (75.4%)	266 (24.6%)	0.81	0.72 to 0.90
**Chinese**	74 (76.3%)	23 (23.7%)	0.77	0.53 to 1.09	69 (79.3%)	18 (20.7%)	0.68	0.44 to 1.01
**Mixed**	2,635 (71.3%)	1,060 (28.7%)	0.94	0.89 to 0.99	1,941 (71.1%)	790 (28.9%)	0.95	0.89 to 1.02
**Other**	711 (81.9%)	157 (18.1%)	0.59	0.51 to 0.68	976 (78.8%)	263 (21.2%)	0.70	0.62 to 0.78
**Missing**	660 (80.8%)	157 (19.2%)	0.62	0.54 to 0.72	660 (80.8%)	157 (19.2%)	0.63	0.54 to 0.73

Note: n=30,426. The n=817 in the missing ethnicity group are those with ethnicity data available neither in CRIS, nor the NPD.
Abbreviations: CI=Confidence Interval, CRIS=Clinical Record Interactive Search, NPD=National Pupil Database, RR=Risk Ratio.

For peer review only

Table S8: Ethnicity overview after supplementing missing ethnicity data from one source with ethnicity data from the other source.

		n (%)
**Usable NPD-derived ethnicity, supplemented from CRIS if missing, n=29,609**		
	White	15,978 (54.0%)
	Black	7,935 (26.8%)
	Asian	1,036 (3.5%)
	Chinese	97 (0.3%)
	Mixed	3,695 (12.5%)
	Other	868 (2.9%)
**Usable CRIS-derived ethnicity, supplemented from the NPD if missing, n=29,609**		
	White	16,324 (55.1%)
	Black	8,146 (27.5%)
	Asian	1,082 (3.7%)
	Chinese	87 (0.3%)
	Mixed	2,731 (9.2%)
	Other	1,239 (4.2%)

For peer review only
